# A secure and decentralized SSI authentication protocol with privacy protection and fine-grained access control based on federated blockchain

**DOI:** 10.1371/journal.pone.0274748

**Published:** 2022-09-23

**Authors:** Binhao Ma, Xurui Zheng, Can Zhao, Yibing Wang, Dejun Wang, Bo Meng

**Affiliations:** School of Computer Science, South-Central Min Zu University, Wuhan, China; University College of Engineering Tindivanam, INDIA

## Abstract

Self-sovereign identity authentication protocol is an active research topic in the field of identity authentication and management. However, the current SSI authentication protocols pay little attention to privacy protection and the fine-grained access control. Therefore, a secure and decentralized SSI authentication protocol with privacy protection and fine-grained access control is proposed. Firstly, the formal model of SSI including the SDPP-SSI identity model and management model is presented. And then, based on the federated blockchain, the distributed identifier is used as a global identifier for users in the decentralized domain. Finally, the verifiable statement is encapsulated using a policy control signature supporting privacy protection to develop the user’s access control for identity registration in the centralized domain. Compared with the related work (Lin 2018, Zhu 2018, Stokkink 2018, Hammudoglu 2017, Othman 2017, Abraham 2018, Guan 2019, Lin 2019) from controllability, security, flexibility, privacy protection, authentication and fine-grained access control, the proposed SSI authentication protocol not only meets controllability, authentication, and flexibility, but also supports privacy protection and fine-grained access control.

## Introduction

Traditional digital identity stored in a centralized database in a unified manner has the risk of information leakage of identity information. But Self-Sovereign Identity (SSI) authentication protocols [1–3] were designed to not only make users control personal identity information but also to be no need for a central trusted authority. Users store their identity data locally on the device and provide the required information to those who need it for verification. With the development of blockchains [4, 5], blockchain-based SSI authentication protocols [6–10]were proposed recently. In 2018, Gräther et al. [6] applied blockchain-based SSI to education certificate management to prevent education certificate forgery; Soltani et al. [7] proposed a self-sovereign identity model for users onboarding on blockchain. In 2021, Abraham et al. [8] introduced a SSI model utilizing identity wallets to ensure that the identity data control remains with the related user; Aruna et al. [9] used a secure data migration to and from the cloud using SSI which facilitates peer-to-peer transactions; Figueroa-Lorenzo et al. [10] presented a solution based on self-sovereign identity on hyperledger fabric blockchain. However, these current SSI authentication protocols pay little attention to privacy protection and the fine-grained access control of identity information.

Therefore, this paper proposes a secure and decentralized SSI authentication protocol which not only meets controllability, authentication, and flexibility, but also supports privacy protection and fine-grained access control of identity information. The main contribution of this paper is as follows.

1. Present a formal model of SSI including secure and decentralized SSI Authentication protocol with privacy protection identity (SDPP-SSI) model and SDPP-SSI management model. And then divide SSI management model into centralized and decentralized types.
2. Propose a secure and decentralized SSI authentication protocol with privacy protection and fine-grained access control of identity information, in which distributed identifiers are used as global identifiers in decentralized domains based on federated chains, policy control signatures supporting privacy protection proposed by us in [11] is used to encapsulates verifiable statements to achieve user access control for identity registration information in centralized domains.
3. Analyze the proposed SDPP-SSI authentication protocol and the results show that it not only satisfies fine-grained access control and privacy protection but also supports controllability authentication, and flexibility. The authentication and privacy are verified automatically using the Applied PI calculus and ProVerif tool.
4. Compared with the related work [12–19] from controllability, security, flexibility, privacy protection, authentication and fine-grained access control, the proposed SSI authentication protocol not only meets controllability, authentication, and flexibility, but also supports privacy protection and fine-grained access control of identity information.

## Related work

The research on self-sovereignty can be divided into three types: SSI’s development, blockchain-based SSI system and blockchain-based information security system. In this section, the related work on the three types are discussed.

### SSI’s development

As society becomes more connected and digital, the number of systems and identities that need to be managed increases significantly. So designing next-generation identity management frameworks quickly is on the agenda. In 2005, Cameron [20] described seven requirements for the success and failure of digital identity systems and was one of the first to explore similar concepts for SSI. SSI can eliminate the need for a centralized trust authority, where users can store their identity information locally on their devices and provide the required information to those who need to authenticate [21]. In 2016, Allen [22] used these seven requirements to list ten principles for industrial SSI, and these ten principles focus on user controllability. Bitcoin has played an important role in SSI evolution because it supported for distributed ledger technology [14]. To address the requirement that identity information in SSI belongs only to the user, a mobile SSI authentication system that relies only on local processing of biometric-based features was designed [15]. The controllability and security of SSI are satisfied, but the biometric data is stored on the blockchain-associated media, which can be accessed by anyone and the user’s private information can be easily leaked.

A large body of literature has introduced various new identity digital ID identifiers based on SSI. In 2018, Electronic Identities (EID) [17], an SSI digital identity,was introduced using distributed ledger-based technology. Using a data format based on Distributed identities (DIDs) and Verifiable Claim (VC), the identity information is asserted and a revocation mechanism is introduced to ensure the validity of the data, but it lacks fine-grained access control on identity information to ensure privacy during authentication, and it is only for one system, which lacks the portability of SSI. In 2016, the W3C organization [23] created a form of WebID identity that implements global identification and authentication in a distributed manner by combining linked data and asymmetric cryptography. While the identity controllability of SSI is satisfied, both the public key and the WebID are tightly bound to the configuration-oriented document describing the authentication, stored in the same third-party server as the OpenID.

### Blockchain-based SSI system

As a new generation of authentication concepts, many enterprises are trying to develop SSI identity systems. And most of the SSI systems based on blockchain are still in testing. Although blockchain technology can provide various technical advantages, there are still some shortcomings for achieving fine-grained authorization and privacy protection of SSI.

Uport [24] is a distributed identity system that supports the concept of SSI identities and runs mainly on the Ethereum. Uport allows only one identity to be created, so users cannot create identities on demand and Uport does not support full autonomy of identities. Jolocom [25] is similar to Uport and is also developed based on Ether and consists mainly of multiple Ether smart contracts. The only distinguishing factor between Jolocom identities and Uport identities is the structure and representation of the identity information, which is not explored further. Sovrin [26] aims primarily to promote the concept of SSI authentication and has developed the Sovrin identity system, which utilizes the Sovrin blockchain and a consensus protocol called Plenum,in which users use mobile applications or websites as Sovrin clients to interact with the distributed ledger in a range of operations such as creating, updating, managing, and sharing their identity information. However, it is still in the development cycle and has a very limited release. Blockcerts [27] uses a blockchain to store and verify the cryptographic hash of any digital certificate. However, unlike Uport, Jolocom, and Sovrin, Blockcerts is not a full-fledged SSI identity system. HomeChain [19] upload its public key and its prepared group signature into the blockchain’s smart contract to anonymously authenticate group members which meets privacy, anonymity, authentication and traceability, but not meets fine-grained access control.

### Blockchain-based information security system

Traditional identity authentication and data transmission schemes often have many security problems. With the development of blockchain-based SSI systems, more and more blockchain-based information security systems have been proposed, BBAAS [28] proposed a blockchain-based anonymous authentication scheme for providing secure communication in VANETs. Bua [29] proposed blockchain to make dispersed SMs constitute a distributed data sharing database. Gupta, Brij B., et al. [30] proposed a truly decentralized, robust and computationally efficient ABSE scheme for healthcare CCPS with the assistance of consortium blockchain. Nguyen, Gia Nhu, et al. [31] developed secure intrusion detection with blockchain based data transmission with the classification model for CPS in the healthcare sector. Lu, Junqing, et al. [32] constructed a secure cloud storage protocol for sensors in IIoT using blockchain technology.

### Problem statement

The problem we address in this paper is that the current SSI authentication protocols pay little attention to privacy protection and the fine-grained access control of identity information, therefore, we proposed a secure and decentralized SSI authentication protocol that supports privacy protection and the fine-grained access control.

## Preliminaries

### DID

The W3C organization has constructed the DIDs scheme [26], which is mainly used as a verifiable identity identifier on a distributed digital identity platform. Compared with PKI-based identity systems, digital identity systems using DIDs have features such as protection of user privacy and security and portability. Its main advantage is that DID is a new type of identity identifier which can be independent of centralized trust institutions, and each user’s identity is controlled by individuals.

### VC

While a declaration is generally an assertion of something by someone, a VC is a partial identity issued by the publisher who proves that users possess certain attributes. This identity can be controlled by the user himself, and any relying party that needs to identify the user will see the user-controlled partial identity associated with him. To make the identity more convincing, the relying party needs to establish a trust relationship with the statement issuer, and the basis of this new relying relationship is the blockchain.

### PCS-PP

The scheme proposed by us in [11] supports fine-grained access control of the authenticator and protects the identity information in the access control policy of the authenticator. The scheme uses LSSS matrices with strong expressiveness to express the access structure and a policy hiding method that exposes public attribute names and hides attribute values using a bilinear group of 3 prime meromorphic orders based on data distortion.

### Federated chain

Federated chain [5] is a system form between the public chain and the private chain, which is often controlled by multiple centers. Several organizations work together to maintain a blockchain, the use of which must be restricted access with permissions, and related information will be protected, such as supply chain institutions or banking consortia. The typical feature of the federated chain is that each node usually has a corresponding entity, and can only join or exit the system with the approval of the alliance. Institutions and organizations of various stakeholders cooperate closely on the blockchain and jointly maintain the healthy and stable development of the system.

## Formalize SDPP-SSI model

In this section, a formal model of the SDPP-SSI including SDPP-SSI identity model and SDPP-SSI management model is presented.

### SSI property

For property of SSI, there exists several classifications. In 2016,Tobin [26] thinked that it has three groups: controllability, security, and portability. In 2018, Reed [14] proposed existence, controllability, accessibility, transparency, persistence, portability, interoperability, consent, data minimization sharing, and protection for SSI. To avoid disagreement, in 2019, Ferdous [33] provided a comprehensive analysis for the property by distilling the attributes that do not belong to the categories and creating an extended classification of base attributes, security, controllability, flexibility, and persistence. In summary, it can be seen that several cross-properties exist in each category and are interconnected. The main properties presented in Table 1 are of three types: security, controllability, and flexibility.

**Table 1:** SSI property.

Category	Property
Security	Protective,persistent,availability
Controllability	Optional,disclosure,consent
Flexibility	Portability,interoperability,minimum

### Symbols

Symbols and its meaning of SDPP-SSI Identity model are illustrated in Table 2.

**Table 2:** Symbol and meaning.

Symbol	Meaning
*D*	Set of domains
*d* ∈ *D*	The domain of a single organization
*U*_*d*_	Set of user identities in domain *d*
*u* ∈ *U*_*d*_	A user identity of *U*_*d*_
*A*_*d*_	A set of attributes in domain *d*
*i* ∈ *A*_*d*_	A unique identifier attribute for a user’s attributes in domain *d*
*AV*_*d*_	A set of attribute values in domain *d*
*v* ∈ *AV*_*d*_	A attribute value of *AV*_*d*_
*D* ^*dec*^	Decentralized domain Set
*d*^*dec*^ ∈ *D*^*dec*^	A single decentralized domain
*U*_*dec*_	Set of user identities in a single distributed domain
*u*_*dec*_ ∈ *U*_*dec*_	A user identity of *U*_*dec*_
$part_{d}^{u}$	A collection of different attributes for a user in the same central domain d
*ident* ^*uC*^	A user full identity for central domain

### SDPP-SSI identity model

**Fig 1:**
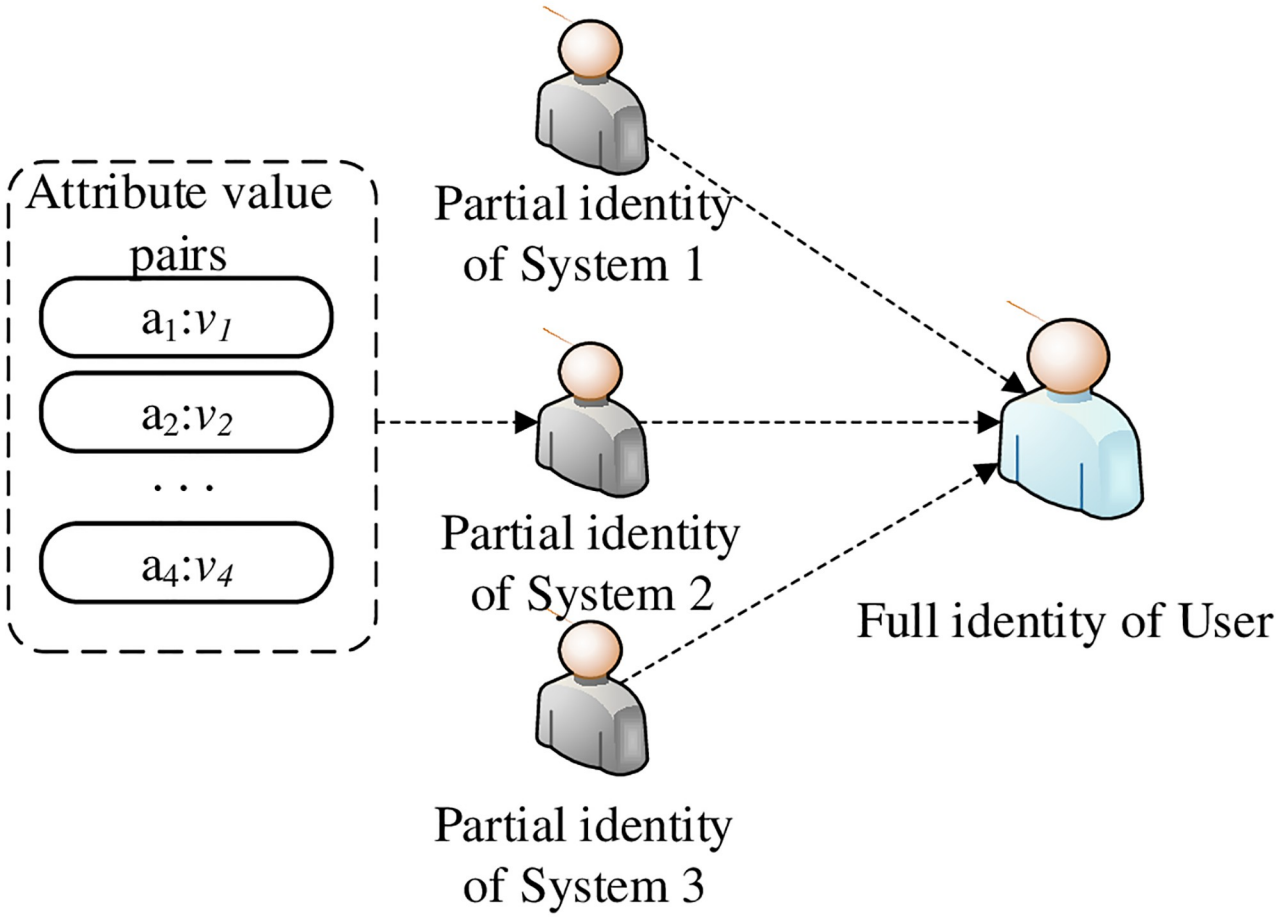
Full identity of user.

**Fig 2:**
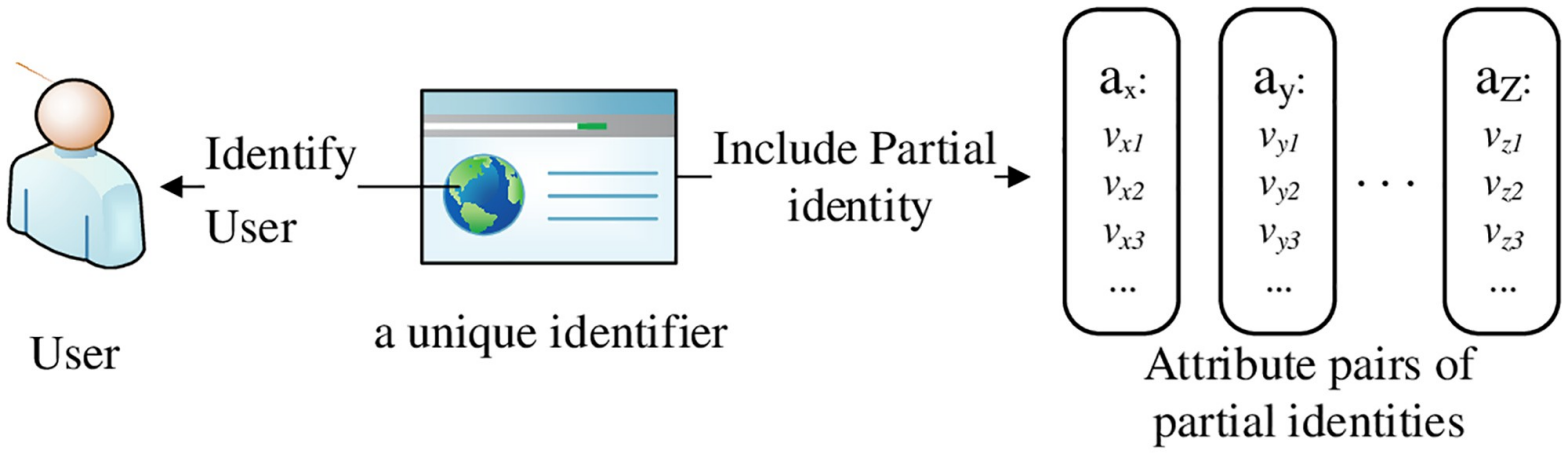
The relationship among a user, a unique identifier and partial identities.

The SDPP-SSI identity needs to be formally defined to eliminate semantic inconsistencies. According to the definition in the digital model [33], the (full) identity of an entity is composed of partial identities in different domains as shown in Fig 1. Partial identities include attributes and attribute values in their domains. The relationship among a user, a unique identifier and partial identities are presented in Fig 2. These partial identities have different authentication function in different entities. The SDPP-SSI identity model mainly includes function ATT, the formal definitions of full identity and partial identities. Function ATT is used to establish the mapping from attributes to attribute values in the identity domain. Based on Function ATT, formal definitions of full identity and partial identities are presented.

**Definition 1:** *ATT*: *A*_*d*_×*U*_*d*_ → *AV*_*d*_: The inputs are the attribute and user identity, the output is the attribute value in the domain *d*.

Function *ATT* produces a corresponding user attribute value with the some user identity attributes in domain *d*. For example, the user want to provide identity attribute (e.g. email, phone number, etc.) in domain *d*, Function *ATT* can generate attribute value. But in other scenarios, for example, the user may not provide some optional identity attributes (such as age, postal address, etc.) with the concerns of privacy, Function *ATT* cannot produce any attribute value.

Function ATT can generate the user attribute value with the following two conditions:

1. If *i* ∈ *A*_*d*_ and *u* ∈ *U*_*d*_,*ATT*(*i*, *u*) = *v*,*v* ∈ *AV*_*d*_
2. For any identities *u*_1_, *u*_2_ ∈ *U*_*d*_, *ATT*(*i*, *u*_1_)≠*ATT*(*i*, *u*_2_).

Intuitively, for condition (1), if a unique identifier attribute *i* for a user’s attributes is in domain *d*, at the same time, *u* is a user identity of *U*_*d*_, function *ATT* must generate the user attribute value *v*.

**Definition 2:** For a user, a full centralized identity is $ident^{uC}=\cup \left\{\left(d,part_{d}^{u}\right)|d\in D,u\in U_{d}\right\}$, in which $\{part_{d}^{u}=\left\{\left(a_{1},v_{1}\right),\left(a_{2},v_{2}\right)...,\left(a_{n-1},v_{n-1}\right)|a\in A_{d},ATT\left(a,u\right)=v\right\}$ are partial identities.

### SDPP-SSI identity management model

**Fig 3:**
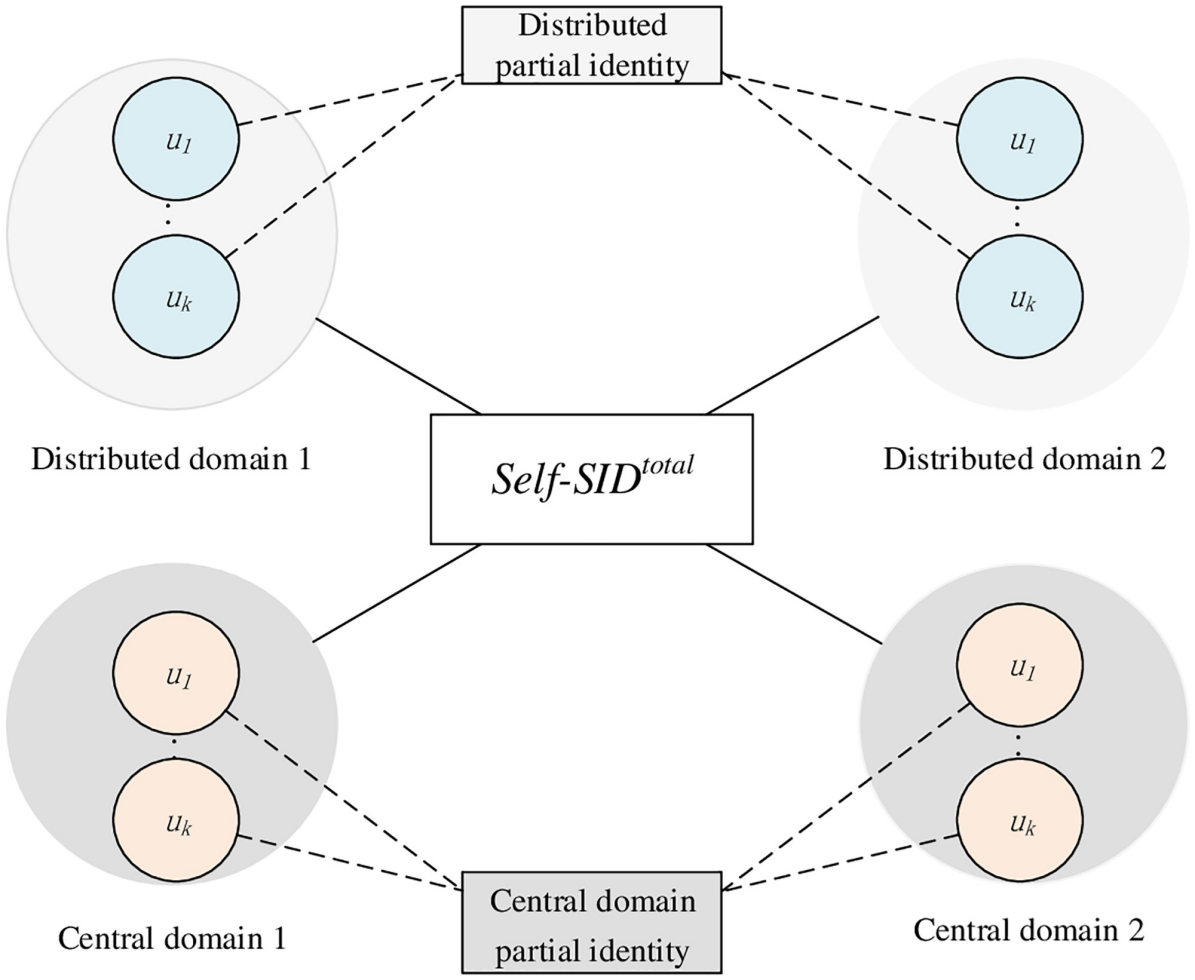
Self-sovereign identity.

Traditional digital identity stored in a centralized database in a unified manner, and people enjoy the convenience while suffering from information leakage. SSI authentication protocols are designed to not only make users control personal identity information but also eliminate the need for a central trusted authority. Users store their identity data locally on the device and provide the required information to those who need it for verification. A distributed domain is an application identity domain that is not controlled and managed by an entity, which allows any entity, including users or providers, participates independently in activities without relying on third party. But if a self-sovereign identity is only composed of distributed domain identities, it is not practical because in practical applications, user partial identities have to rely on Identity Provider (IdP), such as government, financial institutions, etc. Hence, a self-sovereign identity in Fig 3 should be composed of distributed domain identities and central domain.

**Definition 3:** A self-sovereign identity can be formally defined as *Self*−*SID*^*total*^ = *Self*−*SID*^*uD*^∪*Ident*^*uC*^, in which $Self-SID^{uD}=\cup \left\{\left(d^{dec},part_{d^{dec}}^{u_{dec_{k}}}\right)|d^{dec}\in D^{dec},u_{dec_{k}}\in U_{dec}\text{and}k\in Z^{+}\right\}$ is a user full identity for distributed domain and *Ident*^*uC*^ is a user full identity for central domain.$part_{d^{dec_{m}}}^{u_{dec_{k}}}$ are partial identities for a user. If a user has only one identity u, partial identities with the differents *d*^*dec*^ are $\left\{part_{d^{dec_{1}}}^{u_{dec}},part_{d^{dec_{2}}}^{u_{dec}},part_{d^{dec_{3}}}^{u_{dec}}..part_{d^{dec_{m}}}^{u_{dec}}|m\in Z^{+}\right\}$. If a user has different identities *u*_*i*_ $\{u_{dec_{1}},u_{dec_{2}},u_{dec_{3}}.....,u_{dec_{k}}|k\in Z^{+}$ and $u_{dec_{k}}\in U_{dec}\}$, partial identities with the same *d*^*dec*^ are $\left\{part_{d^{dec}}^{u_{dec_{1}}},part_{d^{dec}}^{u_{dec_{2}}},part_{d^{dec}}^{u_{dec_{3}}}...part_{d^{dec}}^{u_{dec_{k}}}|k\in Z^{{}^{+}}\right\}$, and partial identities with the differents *d*^*dec*^ are $\left\{part_{d^{dec_{1}}}^{u_{dec_{1}}},part_{d^{dec_{2}}}^{u_{dec_{2}}},part_{d^{dec_{3}}}^{u_{dec_{3}}},...,part_{d^{dec_{m}}}^{u_{dec_{k}}}|m\in Z^{+},u_{dec_{k}}\in U_{dec}\;\text{and}\;k\in Z^{+}\right\}$.

**Fig 4:**
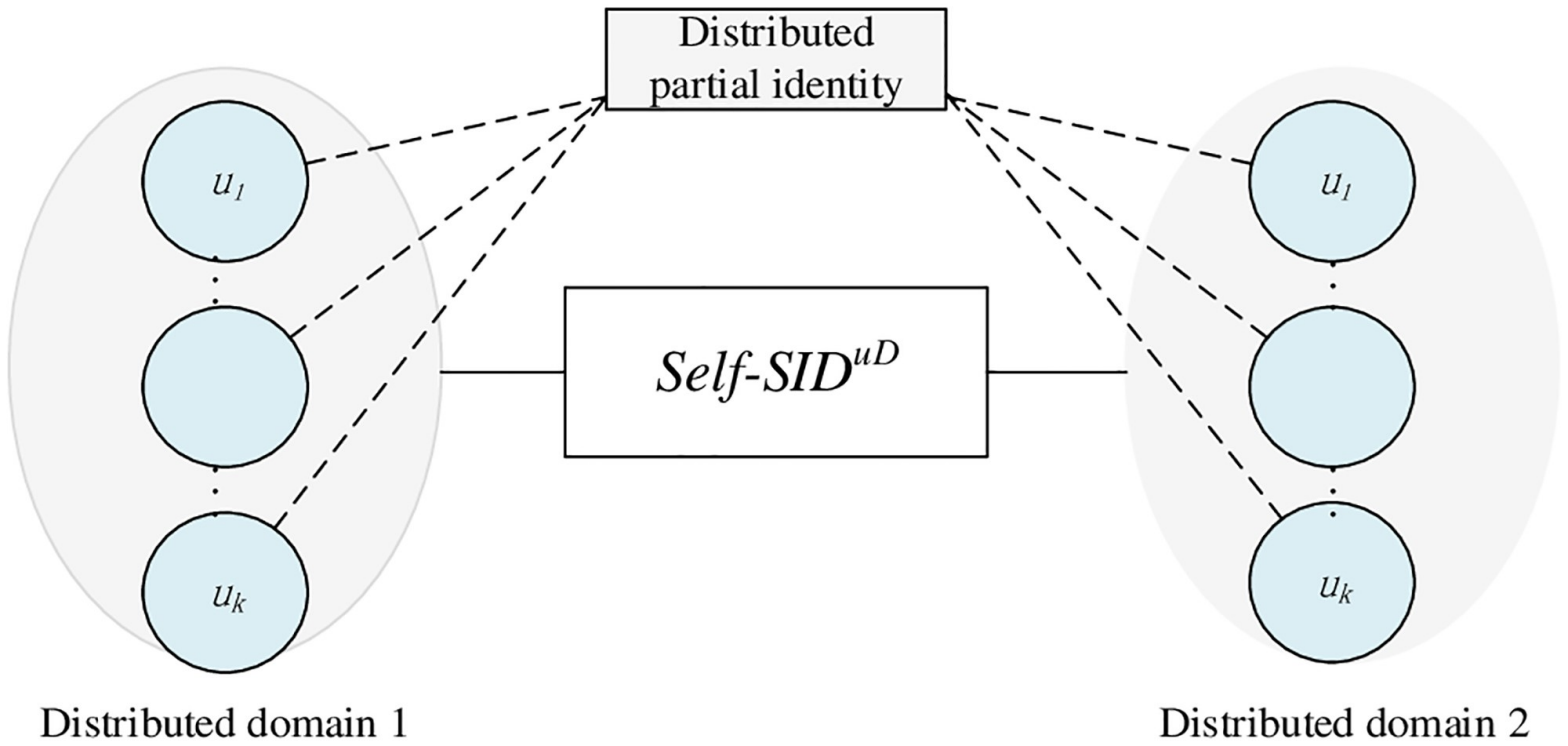
User partial identities distributed domain.

According to the practical requirements, a user may has many partial identities in the distributed domain in Fig 4.

## Framework of SDPP-SSI authentication

**Fig 5:**
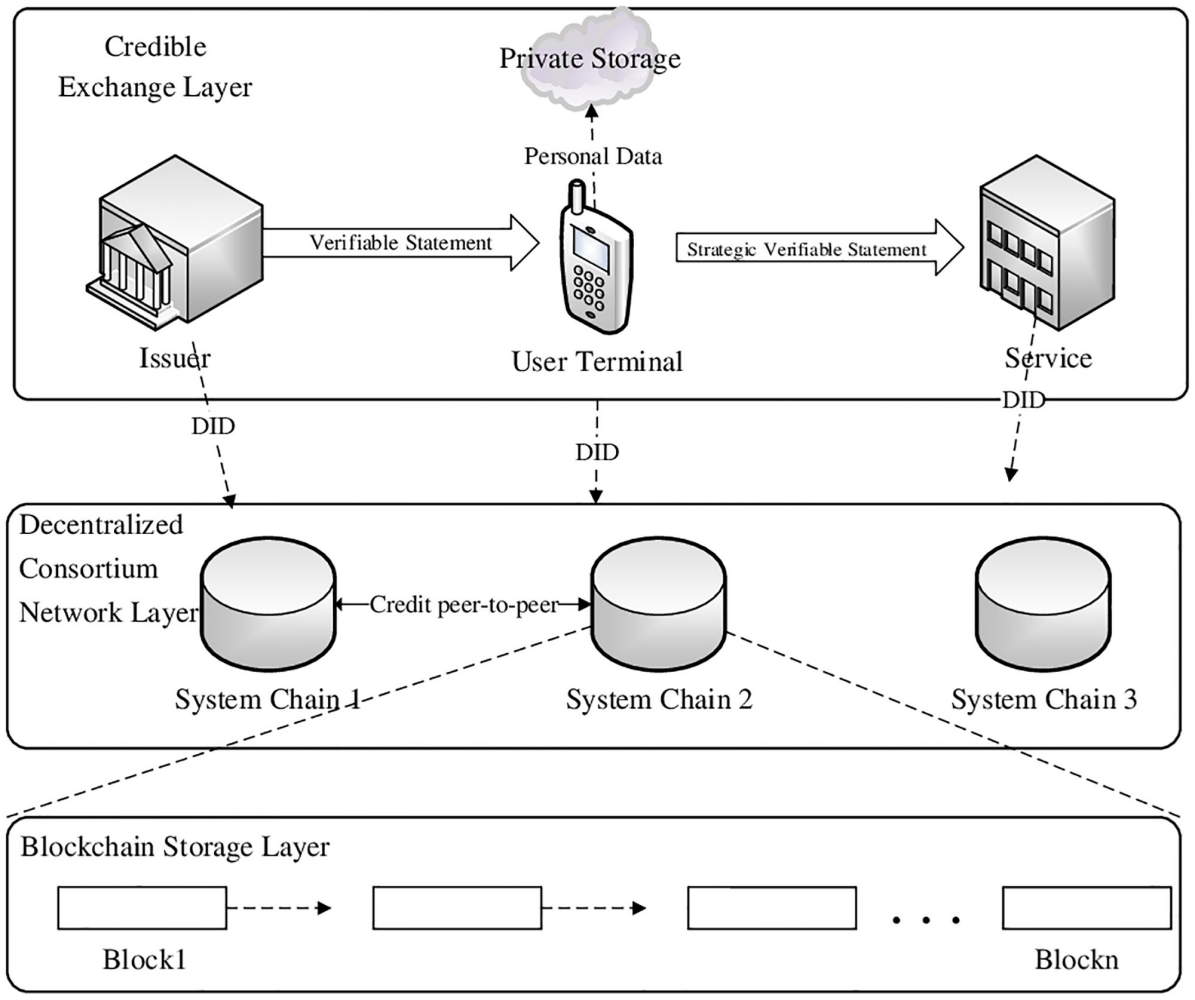
Framework of SDPP-SSI authentication.

In this section, we design a SDPP-SSI authentication framework based on a Fabric federation chain combined with DID, in which PCS-PP wrapper VC is used to generate a Policy Verifiable Claim (PVC). In Fig 5, we divide the framework into three levels: blockchain storage layer, decentralized consortium network layer, and credible exchange layer.

1. *Blockchain storage layer*: The blockchain storage layer is the basic device in the framework, providing trust endorsement for the whole authentication system and acting as a trust bridge between the user and the server. It mainly stores the DID and DID documents, while the user public key and other authentication components are stored in the document. The server get the DID document through the DID resolver of the node to authenticate. Using different federated chain nodes, blockchains in different security domains can be accessed and parsed. Based on federated chains, each chain is accompanied by a data structure called world-state, which is used to keep the current state of the ledger data. The validity of the data can be checked by directly viewing the world-state.
2. *Decentralized consortium network layer*: The federated chain network has multiple organization nodes for multiple domains, and each organization node is responsible for packaging and parsing the relevant requests from different user nodes. Users cannot operate the federation chain state directly, but can only process or return data after authorizing access to different domains and submitting the data or requests to the organization nodes in the domain.
3. *Credible exchange layer*: Credible exchange layer is the information exchange layer, in which the various eco-participants in the SSI authentication system establish secure identities with each other. It can be classified into centralized and decentralized identities according to the scope and role of identity requirements by service providers. Users need not to provide detailed identity information, but only need a valid DID for authentication login, which is generated by users themselves in a decentralized domain.

## The SDPP-SSI authentication protocol

The proposed SDPP-SSI authentication protocol contains identity registration, identity authentication, and identity revocation processes. Each of them is further divided into the interaction process between two identity domain types: central and distributed domains. The roles include user, organization node, blockchain, state of the world, IPFS, trusted third party, and server. The organization node is assigned permissions by domains that can be based on users. And the state of the world is used to record whether the data is valid or not.

### Enrollment process

Identity information contains both traditional central domain identity and distributed domain identity. So there are two cases about defining identity registration, one is to directly make a declarative assertion of one’s identity and generate a DID, without the need for a third party in the central domain to prove the attributes owned by one’s identity. This kind of identity is suitable for fast authentication scenarios in distributed domains that do not need to provide detailed proofs, such as registering a login for a common website. Another type of identity registration requires proof of one’s identity by a third party in a traditional central domain to generate signature-like VCs that are used to authenticate services that require identity attributes to achieve certain privileges.

#### Distributed domain registration

**Fig 6:**
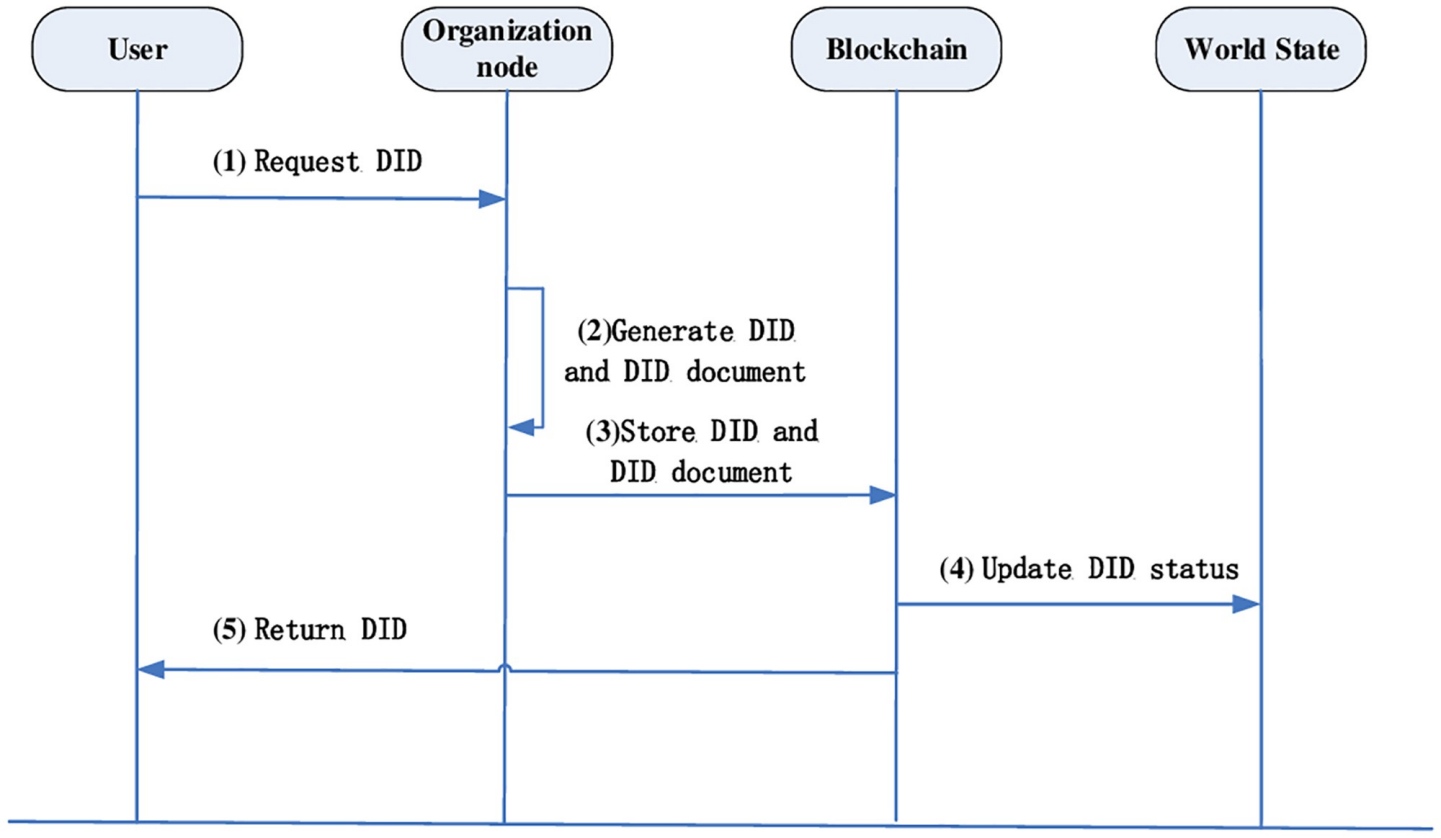
Distributed domain registration process.

Since third party is not required to provide proof of identity, in the registration phase, entity contains only the user and the blockchain. Based on the federated chain network, each blockchain with different identity domains has a dedicated organization node to process the data requests submitted by the user end nodes. After the organization node processes the data, there is a world state to keep the latest state of the DID if it involves reading and writing of the same DID. The flowchart of distributed domain registration is shown in Fig 6.

1. The user generates a request to create a DID. First, the user generates a random seed to generate a public-private key pair <SK, PK >, saves the private key SK locally, and then selects the federated chain node that needs to be registered, and the user sends the public key PK to request the generation of a DID.
2. The federated chain node receives the request in the DID generation algorithm in Table 3, and generates DIDC according to the user’s PK by the DID generation algorithm. The document stores the user’s public key, the port of the federated chain node and other authentication information, and uses the double hash algorithm to generate DIDC into DIDs, which can be parsed as DIDC and recorded in the registry.
3. Store DIDs and DIDC as key-value pairs in a blockchain within the domain.
4. Update the world state about this DID to be valid with the latest state.
5. Return the DID to the user terminal and complete the decentralized domain registration after local storage.

**Table 3:** DID generation algorithm.

Algorithm DID Generation
Input: *PK*_*U*_ Output: (*DID*,*DIDC*) or False 1: *DIDC* = SetDIDC (*PK*_*U*_) 2: *DID*=did——method——*Base*58(*ripemd*160(*sha*256(*DIDC*))) 3: if *DID*=Null 4: return False 5: else if 6: return (*DID*, *DIDC*) 7: end if

#### Central domain registration

**Fig 7:**
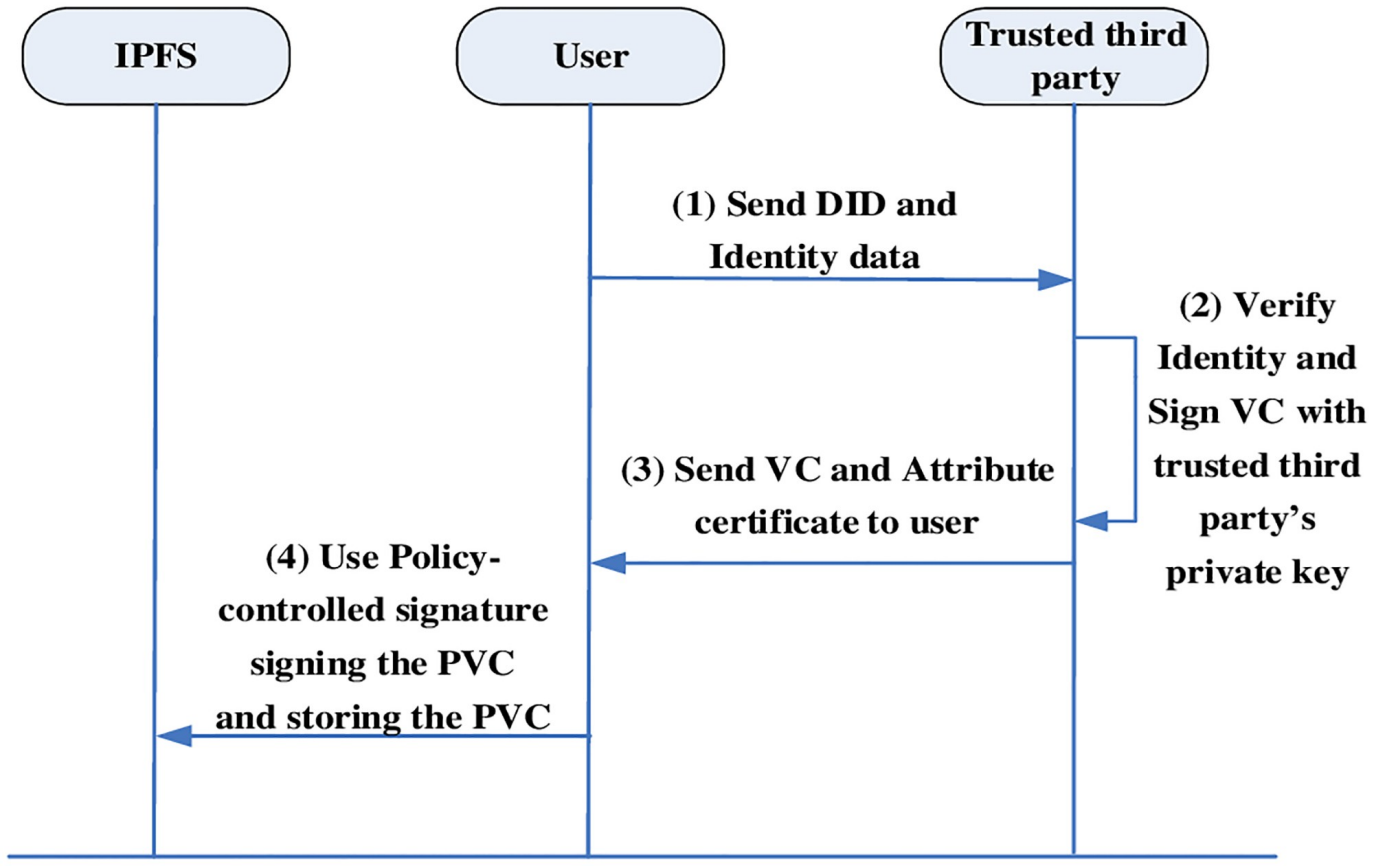
Central domain registration process.

The central domain registration generates VC for the user’s DID, and the user submits the required Identity information to the trusted third party, after confirming the identity on other official platforms outside the chain, uses the private key paired with the public key in the third party’s DID document to sign the user’s identity to get the verifiable statement VC. after returning the VC to the user, the user uses the policy control signature to sign the VC to generate the policy the verifiable statement PVC. The flowchart of central domain registration is shown in Fig 7.

1. Users enters personal identifiable attributes and digital assets, such as age and driver’s license. After that, the DID and the digital identity verified successfully are submitted to a trusted third party in an encrypted channel.
2. The trusted third party confirms the user’s digital identity through external verification, and then the private key SKTA of the trusted third party DID is used to assert that the user satisfies a certain identity and generates a VC, and at the same time, in order to verify the signature of others, an attribute certificate is generated based on the user’s attributes, and the VC and the attribute certificate are sent to the user. For example, if a service requires a person to be at least 18 years old and the user is 23 years old, the assertion is “over 18 years old”.
3. The user receives the verifiable statement VC and the attribute certificate Cre, and then formulates the access policy, and the user signs the access policy to the VC by the policy control signature algorithm to get the policy verifiable statement PVC.
4. User stores PVCs on IPFS or private cloud storage to complete centralized domain registration.

### Authentication process

**Fig 8:**
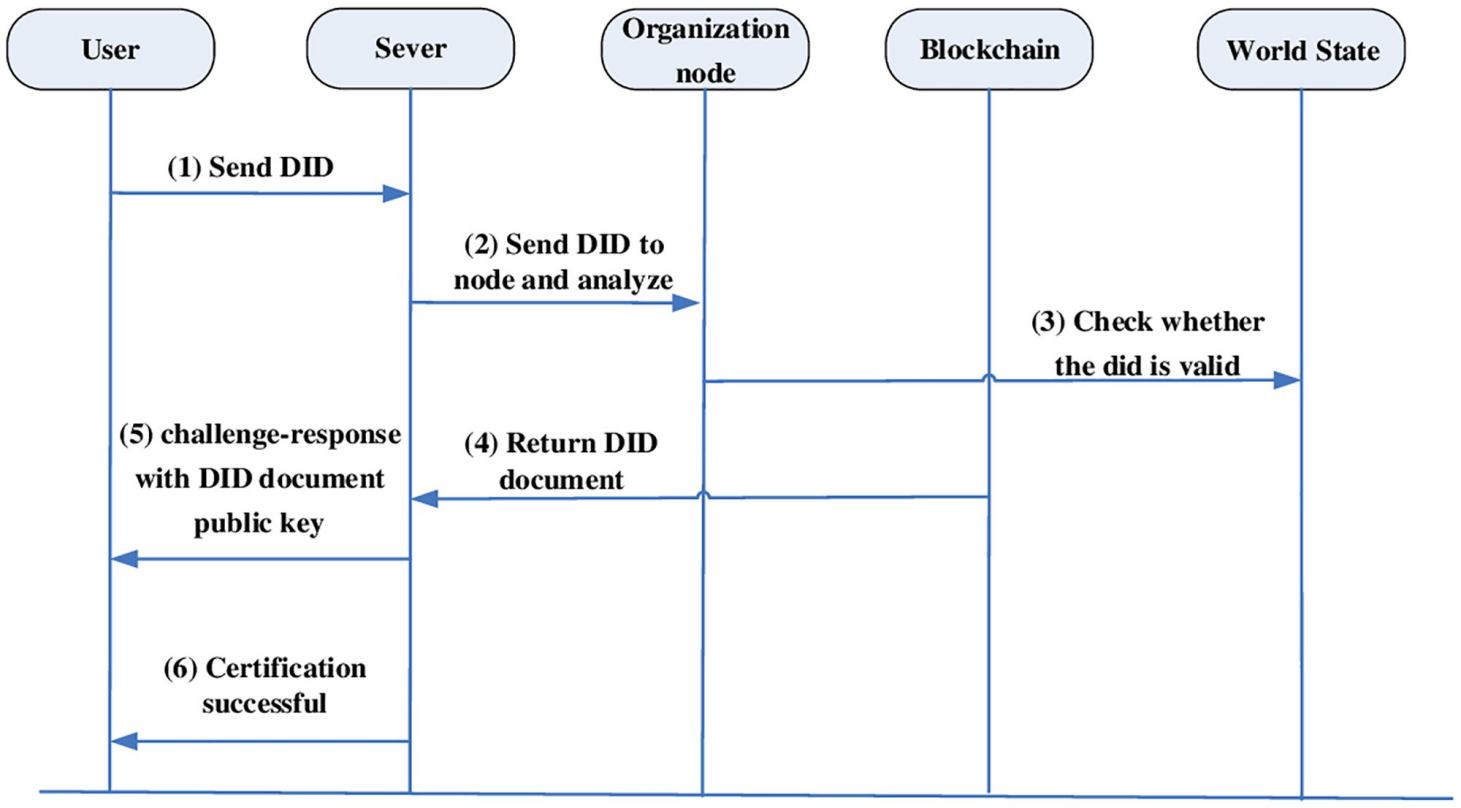
Distributed domain authentication process.

**Fig 9:**
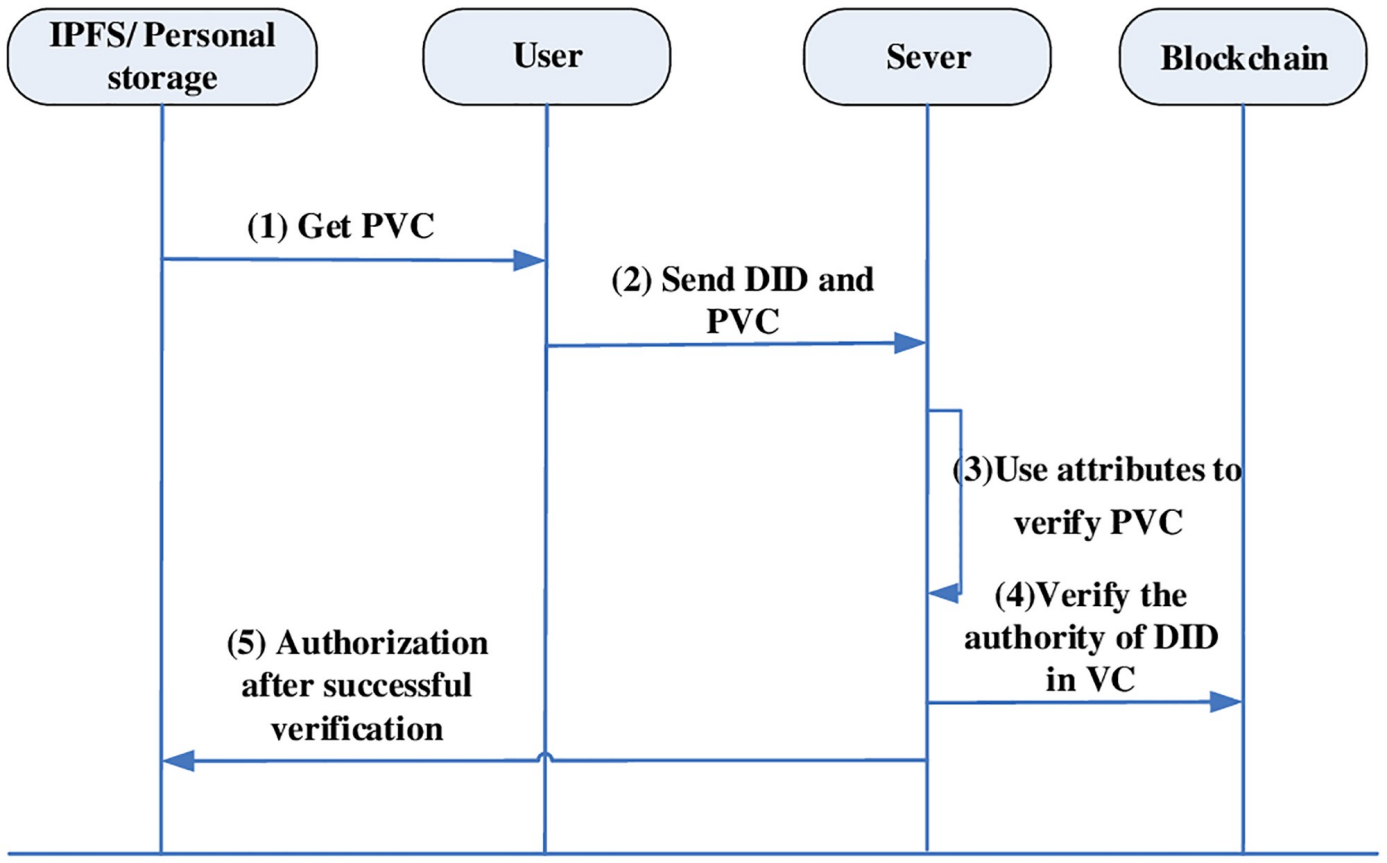
Central domain authentication process.

The authentication process also consists of two types. One uses DID to authenticate its identity by checking the validity of the authentication component in the DIDC in the federated chain to authenticate the DID. The other needs a central domain authenticated VC to prove the attributes possessed by its own identity. As shown in Figs 8 and 9, the distributed authentication process and the central domain authentication process are shown respectively.

#### Distributed domain authentication

1. The user requests the service and sends the personal DID to the server.
2. The server receives the user DID, as shown in the algorithm in Table 4, and according to the services required by the user such as tax check service, he first enters the tax domain, and then parses the DID through the tax domain blockchain organization node to get the DID document.
3. After getting the DID document, first check the world state to check if it is within the validity period.
4. If it is valid, the return state is generated and the information of the DID document is obtained.
5. After the server gets the DID document, it uses the information from the authentication component to run the authentication algorithm for verification. If the public key encryption is used, the authentication algorithm such as encrypting a challenge value nonce with the public key and sending the ciphertext CT to the user.
6. If the verification is valid, the DID login is proved to be successful and the authentication is finished.

**Table 4:** Algorithm DID verification.

Algorithm DID Verification
Input: *DID* Output: True or False 1: s ← False, *Nonce* ← Rand(.) 2: CT= *En* (*Nonce*, *DID*(*PK*_*U*_)) 3: Send *CT* to user 4: PT=*De* (*CT*, *SK*_*U*_) 5: Send PT to Sever 6: if PT =*Nonce* 7: *s* ← True 8: end if 9: return *s*

#### Central domain authentication

The server authenticates the user’s DID through the signature in the PVC. The premise of using VC is that the DID of a trusted third party has been authenticated by the public. However, because the PVC is stored only on the user side and the signature is controlled through the user’s policy, only the service side that satisfies the policy set by the user can successfully verifies the PVC. Ensures the ability of VC to deliver authentication and the controllability of the identity. DID authentication is needed before authentication, and then the identity attribute qualification of the PVC.

1. The user requests the service, the server returns the identity eligibility requirements needed for authentication. The user selects the policy verifiable statement PVC about the identity from the client, and then obtains the PVC from IPFS or the personal cloud.
2. Send the DID and PVC to the server.
3. The server uses Cre, which is its own attribute certificate, to verify the policy-controlled signature on the PVC, and after verifying that the policy is satisfied, the VC is obtained.
4. After the server gets the VC, it first obtains the DID document on the corresponding federated chain according to the trusted third-party DID of the signed VC. if there is no certified third party in advance, it needs distributed domain authentication in Fig 8 to obtain the signature of the user identity statement on the VC to verify the authority. the VC verification algorithm is shown in Table 5.
5. Obtain the authenticity of the VC and authorize the service after successful authentication

**Table 5:** Algorithm VC verification.

Algorithm VC Verification
Input: *DID*_*TTP*_, *POL*, *Cre* Output: True or False 1: *s* ← False 2: *Check*_*pol*_ = (*POL*, *Cre*) 3: if *Check*_*pol*_ ≠ NULL then 4: if DIDVERIFY (*DID*_*TTP*_) ==True then 5: s ← True 6: end if 7: end if 8: return *s*

#### DID revocation

**Fig 10:**
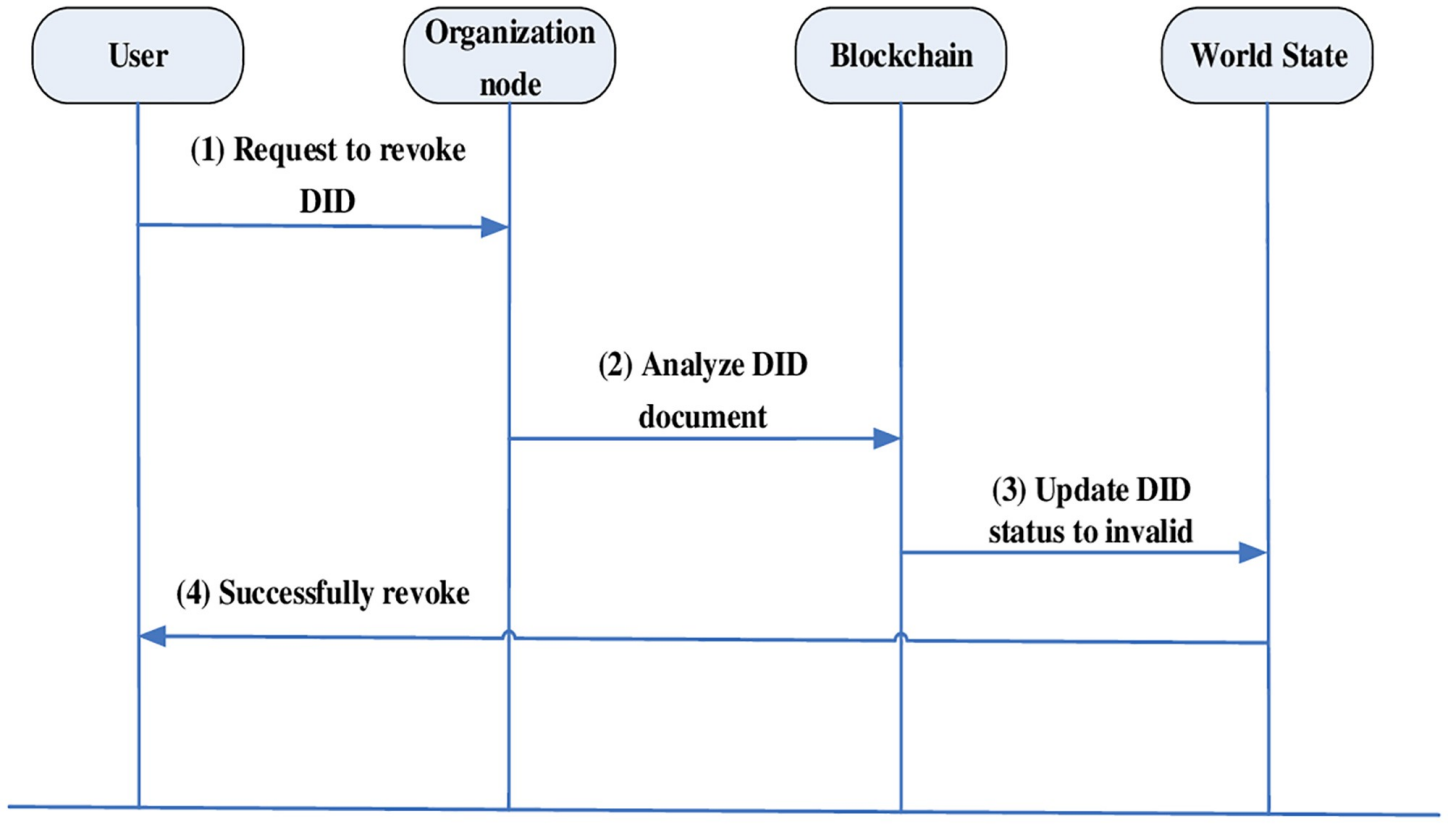
DID revocation process.

Because of tamper-evident feature of blockchain, the revocation of DID can only be accomplished by invalidating the DID recorded on the blockchain. The designed world state not only records the latest state of data, but also records whether the data is revoked or not, so the revocation of DID can be completed by updating the world state. The DID revocation process is shown in Fig 10.

1. The user outputs the DID and submits a DID revocation request.
2. After the organization node receives the DID request, the information is parsed into a DID document.
3. Update the attached DID world state according to the DID document, set the latest DID state to revoked, and record the time.
4. Return state details to user, DID revocation is successful.

#### VC revocation

**Fig 11:**
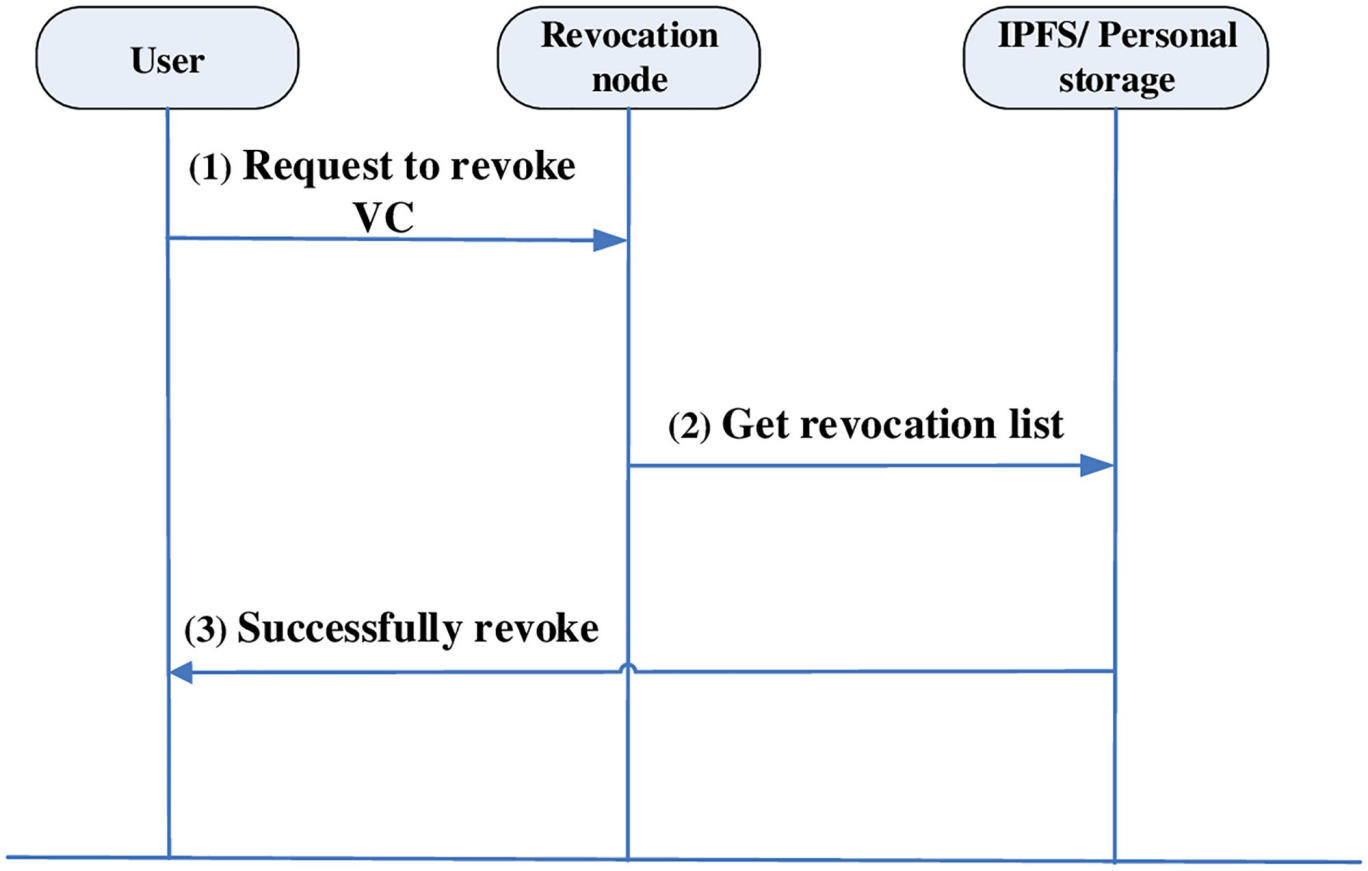
VC revocation process.

The fields for the revoke method are stored in VC. If revocation is required, the DID owner needs to maintain a node that provides revocation itself. When validating the statement, this node need to verify whether the statement is revocated. The VC revocation process is shown in Fig 11.

1. The user removes the PVC and issues a VC revocation request.
2. Get the list of revocations by taking the revocation node from the statement that can check the state of suspensions.
3. The revocation is successful after the revocation node updates the state of the VC in the revocation list to the revocation state and returns the information.

## Property analysis

### Security analysis

The SDPP-SSI authentication protocol designed in this paper differs from other latest blockchain-based identity systems by relying on the federated chain foundation, which allows users to choose different identity domains for authentication, instead of obtaining digital identity information from a third party in the same identity domain. Data consistency is guaranteed by the unforgeability of blockchain. The private data is only stored in the individual user terminal to ensure the personal data security. The combination of policy-controlled signature and VC is also used to ensure fine-grained access to data in the centralized verification process, supporting the privacy and authentication of authentication information. The authentication and privacy are verified formally using the ProVerif tool.

#### ProVerif

ProVerif [34] is an automatic cryptographic protocol verifier based on a representation of the protocol by Horn clauses and the Applied Pi calculus. It can handle many different cryptographic primitives, including shared- and public-key cryptography, hash functions, and Deffie-Hellman key agreements, specified both as rewrite rules and as equations. It can also deal with an unbounded number of sessions of the protocol and an unbounded message space. When ProVerif cannot prove a property, it can reconstruct an attack, that is, an execution trace of the protocol that falsifies the desired property. ProVerif can prove the following properties: secrecy, authentication and more generally correspondence properties, strong secrecy, equivalences between processes that differ only by terms. ProVerif has been tested on protocols of the literature with very encouraging results. When ProVerif cannot prove a security property, it can reconstruct an attack, ProVerif can prove secrecy, authentication, and more generally correspondence properties, strong secrecy, equivalences between processes that differ only by terms.

#### Functions and equation theory

**Fig 12:**
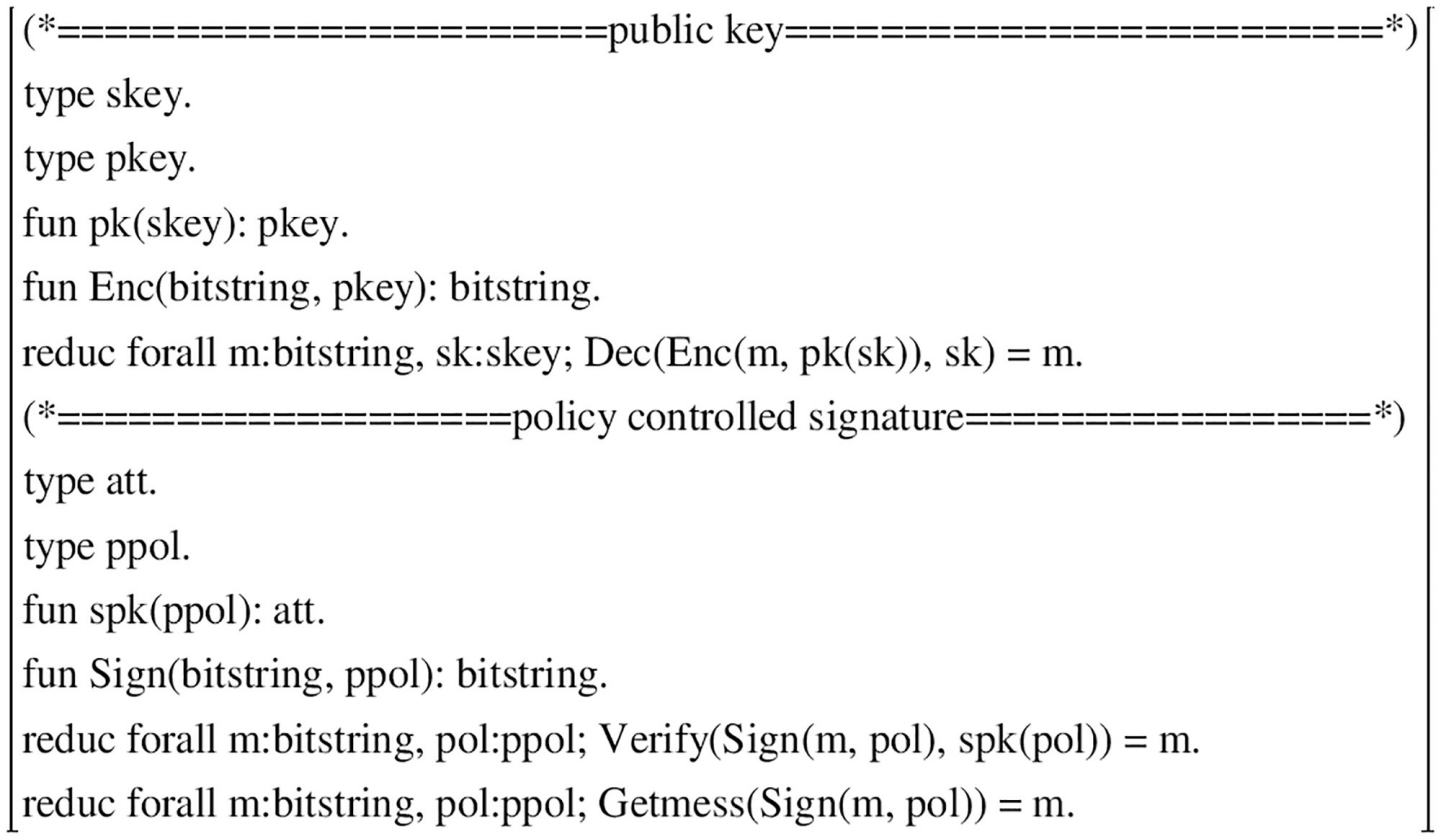
The functions and the equational theory.

Since the cryptographic primitive used for decentralized authentication is the handshake protocol of public-key cryptography, the centralized SSI of the main analysis strategy control signature is used here using ProVerif. First describe the function and equation theory in SDPP-SSI, as shown in Fig 12, the function mainly contains: a pair of public-key cryptography encryption and decryption algorithms Enc(*x*, *PK*) and Dec(*y*, *SK*), policy control signature algorithm Sign(*x*, *POL*), verification algorithm Verify(*y*, *Att*). where the public key cryptography uses decentralized authentication, En(*x*, *PK*) uses the public key to encrypt the challenge value x into ciphertext, De(*y*, *SK*) uses the private key to decrypt the ciphertext y into the challenge value x. The policy control signature can sign the message x using the access structure POL, and only the verifier attribute Att satisfies the access policy POL can verify the signature.

#### Processes

**Fig 13:**
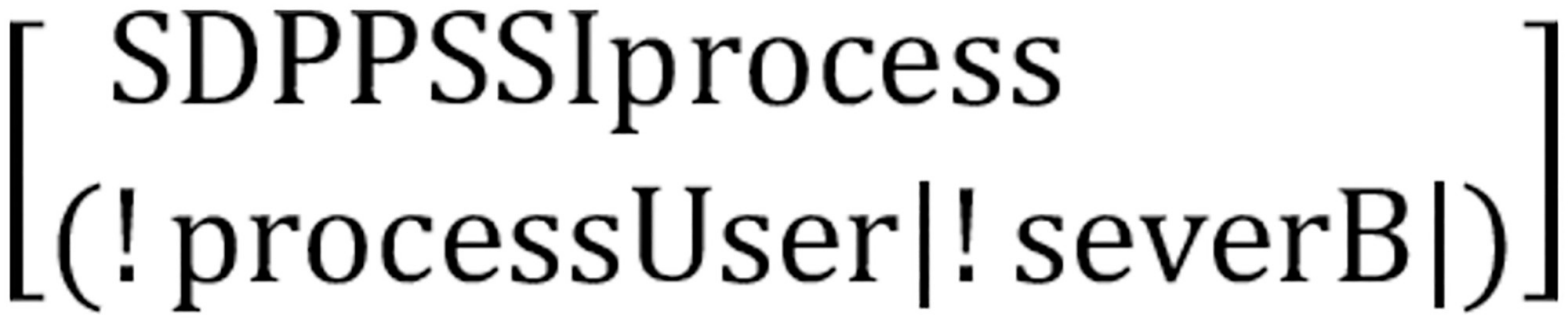
Main process.

As shown in Fig 13, the main process SDPPSSIprocess includes the user process ProcessUser and the server process severB.

**ProcessUser:**The user process mainly completes the identity management in the identity registration phase, and the user submits authentication information in the login phase, etc. Firstly, the registration process of the protocol is modeled by Applied PI algorithm. In the registration phase, firstly, the DID, access policy and timestamp information are entered by new keyword, and then the public-private key pair (*PK*__*U*_, *SK*__*U*_) is generated by invoking the public-key algorithm according to the security parameters of the DID. finally, the DID and *PK*__*U*_ are sent to the authentication server process through the public channel c.

**Fig 14:**
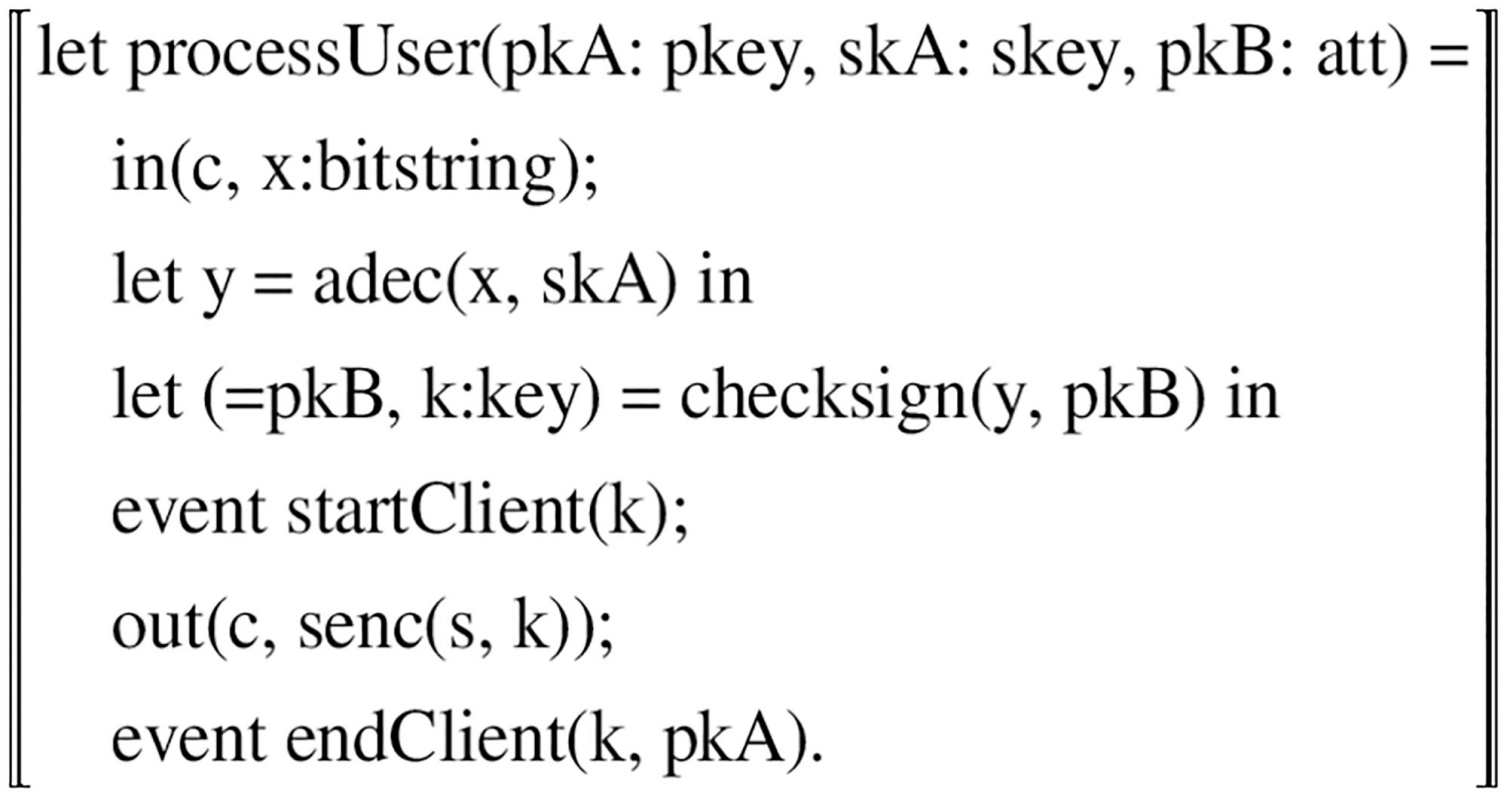
User’s process.

In the login phase, first the user receives the ciphertext challenge value t1 encrypted by the server using the user’s *PK*__*U*_. Then the user decrypts the ciphertext challenge value into a plaintext challenge value using the decryption function dec and his private key *SK*__*U*_. After the user sets the access policy POL, he uses the policy control signature to sign the plaintext challenge value to get the signature. finally, it is sent to the authentication server through the channel c. The detailed representation is shown in Fig 14.

**ProcessServerB:** ServerB, a server-side process, mainly receives user registration information and authenticates users through their requests, etc. In the user registration phase, The server receives the message first binds the message DID and the user public key to get d1. new an event stamp t1, then uses the hash function to calculate the binding value d1 and t1 to get the challenge value c1. use *PK*__*U*_ to encrypt c1 to get the encrypted value s1, and finally send it to the user through the channel.

**Fig 15:**
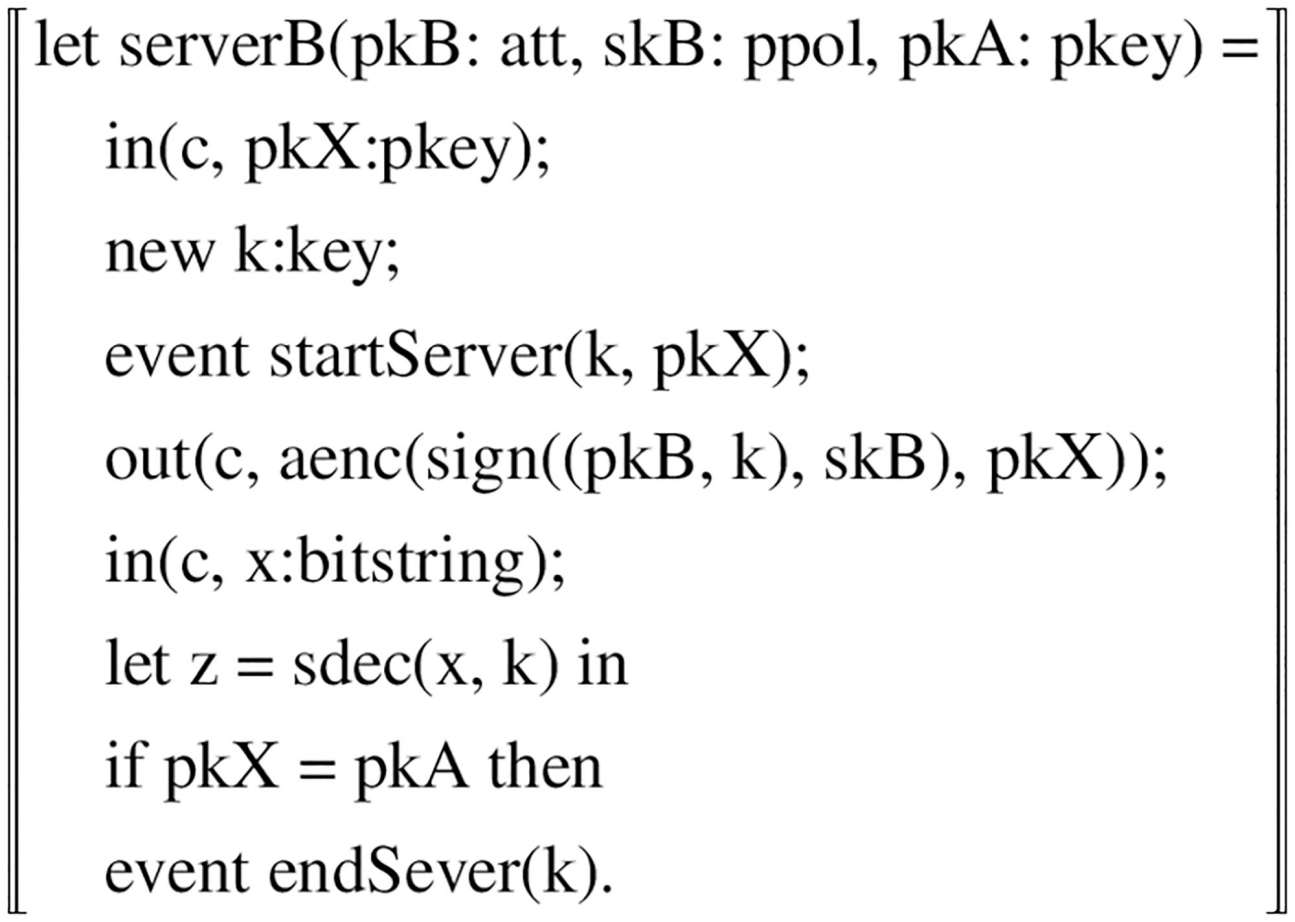
Sever’s process.

In the login phase, the server side receives the message, gets the signature Signature, and then uses the server-side property value attitubes to verify the signature. If the verification fails, The serverhas no authority to verify. otherwise the challenge value plaintext m1 is obtained, and comparing m1 and c1 is equal, the user is said to be a legitimate user. if it is not equal, the login is rejected. In Fig 15 we show the specific representation.

#### Authentication analysis

ProVerif uses the non-injective agreement to model the authentication. So we use query ev: event one ——> ev: event two to model the authentication. It is true when if the event one has been executed, then the event two must have been executed (before the event one). Here we use the non-injective agreement to model the authentications shown in Table 6.

**Fig 16:**
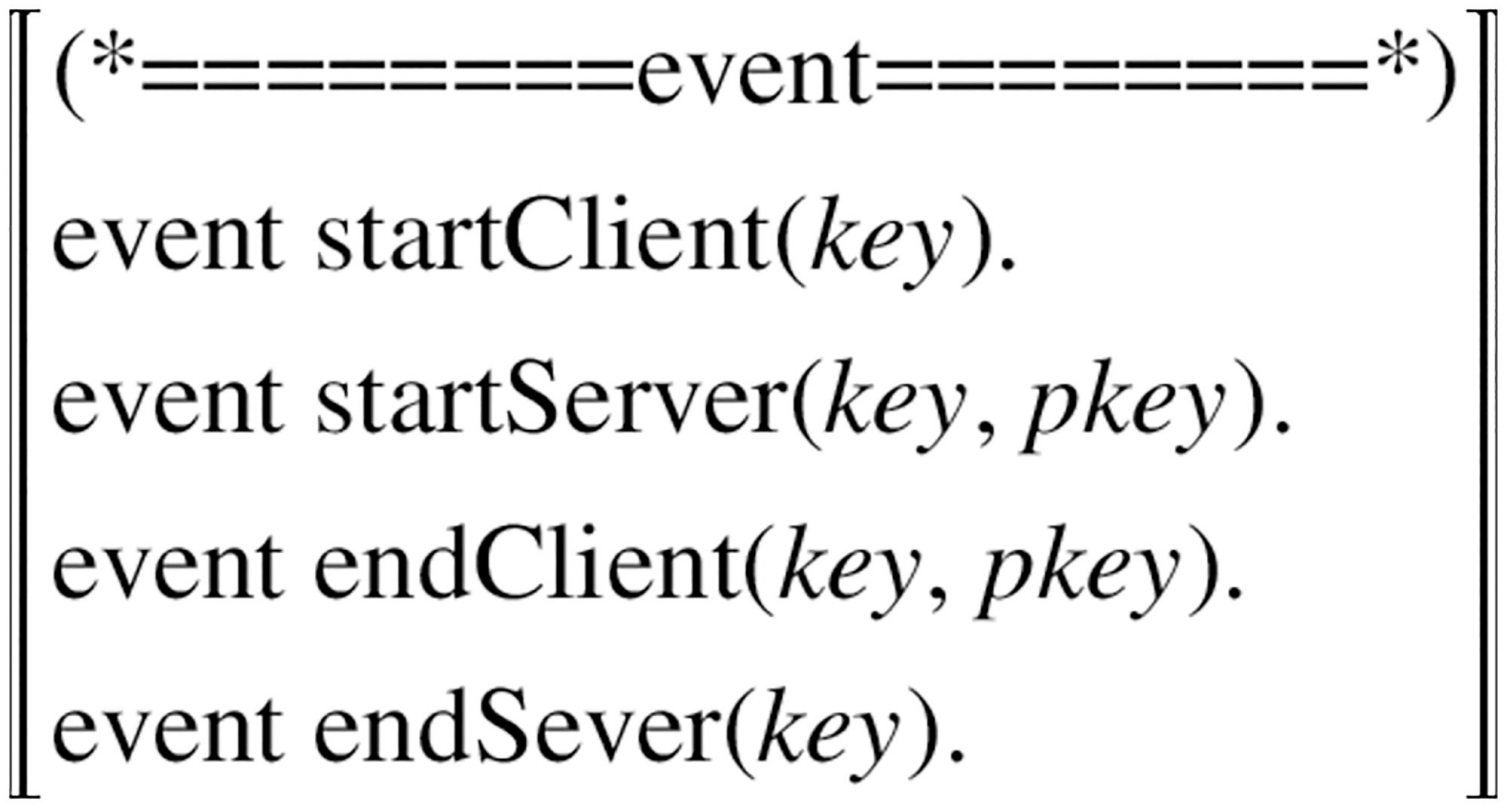
Event.

The events in Fig 16, are the user start, end events, the server start and end events.

**StartClient:** record the receipt of the key provided when running the protocol with the server.
**StartServer:** record the fact that the server has agreed to run the protocol with the client, the first parameter is provided as the key and the second parameter is the client’s public key.
**EndClient:** the client has terminated the protocol using the symmetric key provided as the first parameter and the client public key as the second parameter.
**EndServer:** the server has terminated the protocol running with the client, where the symmetric key is provided as the first parameter.

**Table 6:** The authentications.

Non-injective agreement	Authentications
ev:endClient(key,pkey)→ev:startClient(key)	Identity Provider authecticates User Agent
ev:endSever(Key) → ev:startServer(key,pkey)	Service Provider authecticates User Agent
ev:endSever(key) → ev:startClient(key)	Identity Provider authecticates Service Provider
ev:endClient(key,pkey)→ ev:startServer(key,pkey)	Service Provider authecticates Identity Prvider

**Fig 17:**
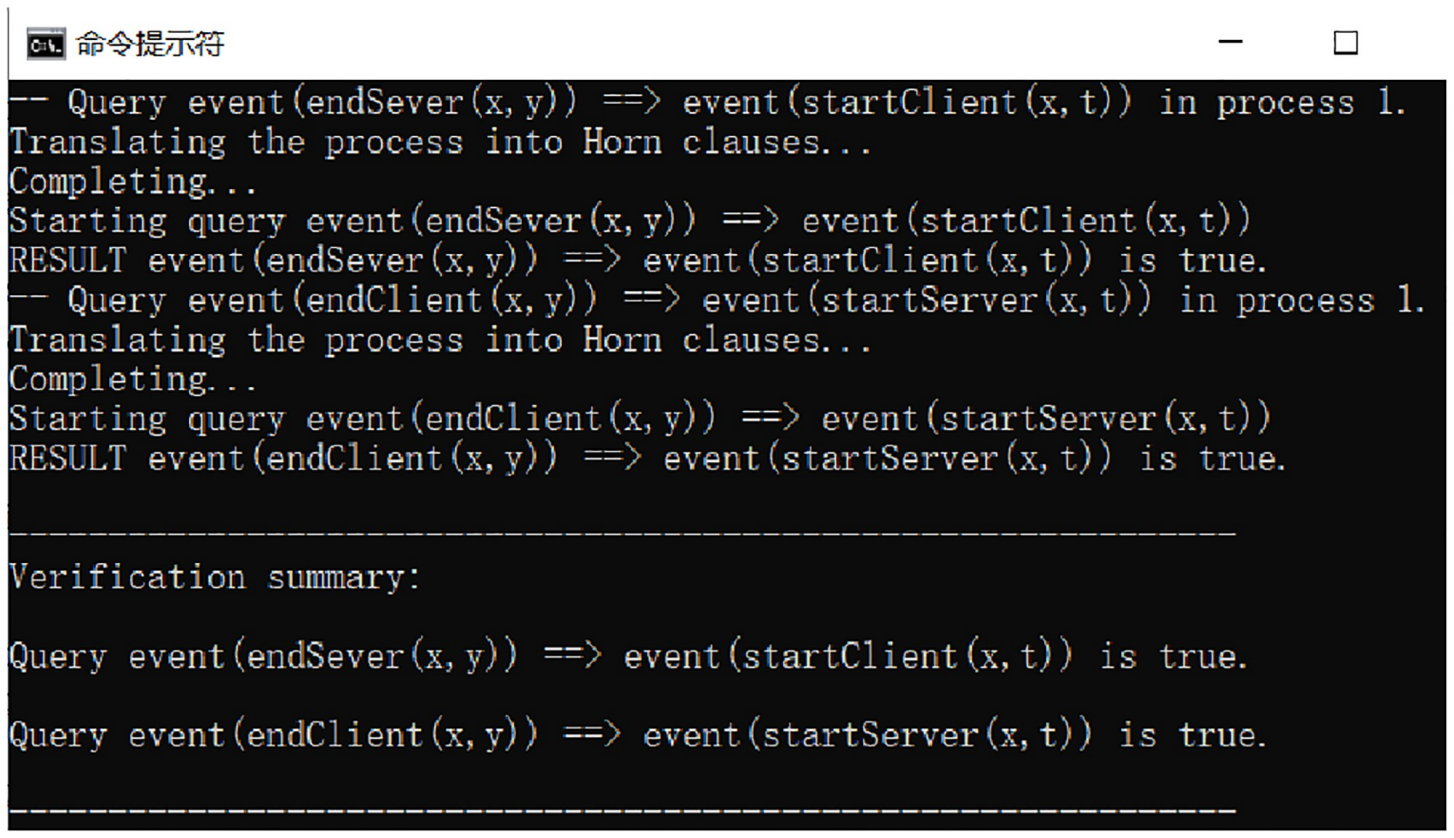
Authentication analysis result.

The analysis result in Fig 17 shows “true”, which proves that the protocol satisfies the authentication of the server to the client. Because the client encapsulates the DID and timestamp and sends the user’s public key to encrypt the information to the user for the challenge answer of authentication, only the user who has the public key corresponding to the private key can decode the plaintext value of the answer. And the user sends the plaintext value to the server after using policy control signature processing, and only the server can get the signed message correctly if it meets the access policy on the signature.

### Privacy analysis

**Fig 18:**
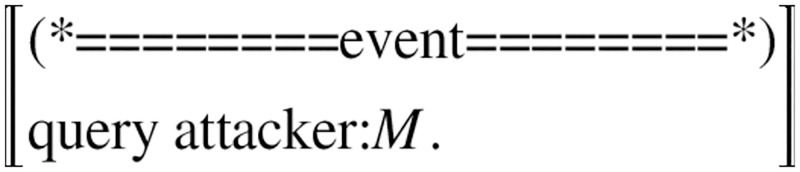
Query event.

The privacy is modelled as the confidentiality of the message *M*. *M* is the private data passed between the user and the server. A query statement about confidentiality on the message *M* is defined in Fig 18.

**Fig 19:**
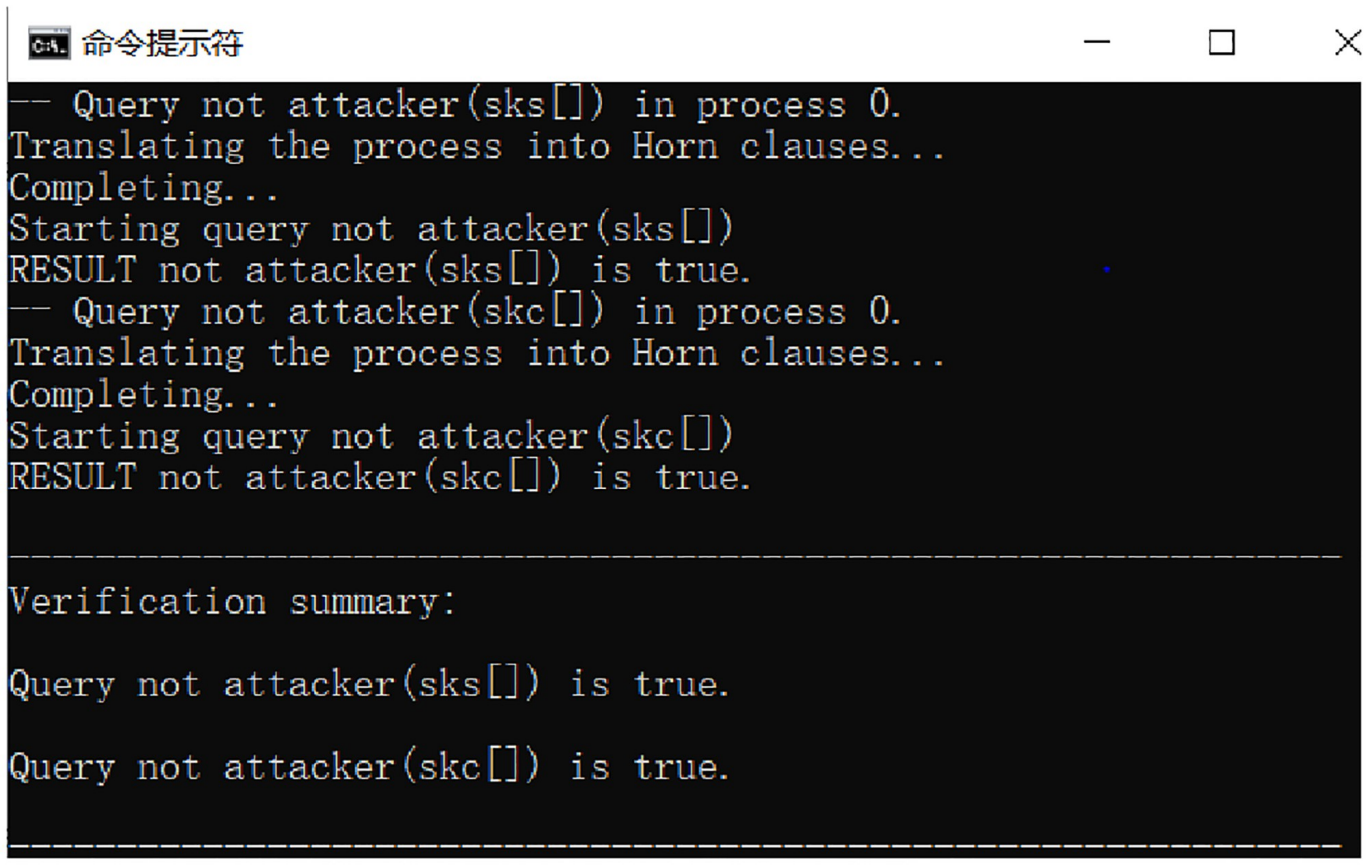
Privacy analysis result.

The analysis results in Fig 19 is “true” which indicates that message *M* has confidentiality during the protocol, and then the model is proved to have privacy. Because the user sends the signature, the message is bound to the DID and timestamp, and then the hash operation is performed to get the password value, and the password value is given to the authentication server attacker if the attacker does not meet the access policy of the signature will not be able to get the correct signature, and will not get the plaintext message.

### Basic property analysis

#### Controllability

The proposed SDPP-SSI authentication protocol relies on the characteristics of the federated chain partly decentralized, distributed, and controllable, which authentication processes belong to different federated domain, giving a reliable base environment for self-sovereignty. The authentication process is divided into centralized authentication and decentralized authentication, and the user and the service can choose different identities to get the corresponding authentication services according to the privacy disclosable principle. Meanwhile, in the centralized authentication process, the fine-grained authentication of digital identity is completed with the proposed policy-controlled signature technology combined with VC. Therefore, users can authenticate in different domains by selecting different authentication processes, achieving controllability in SSI.

#### Fine-grained authorization

The Fine-grained access control is about fine-grained authorization, fine-grained authorization of authenticators is achieved by restricting the authority of authentication signatures by designing privacy-preserving policy control signatures that have different access policies. Based on the federation chain foundation, all the data containing privacy data are stored on the user side, and the chain only stores the DID and DID documents without privacy data. In the authentication process, users can choose to provide different VCs to complete the centralized authentication and implement the fine-grained authorization of Identity information.

#### Flexibility

In contrast to public-chain blockchain authentication platforms that target only one kind of domain, the proposed SDPP-SSI authentication protocol designed in paper uses a federated chain and DID identifier. Users can verify their identities in different domains through different organizational nodes of the federated chain, and the DID information is registered in different domains by the uniqueness of DIDs. The verification of identity information does not depend on the unique identity, but use the combination of multiple DIDs for authentication, and user’s access to the identity information through the authority control of the access policy.

## Comparison and discussion

Comparison and analysis of literature [12–19] and the proposed SDPP-SSI from controllability, security, flexibility,privacy protection, authentication, and fine-grained access control is in Table 7. Reference [12] proposed a blockchain-based secure exchange of biometric credentials SSI authentication scheme. Although the controllability and security of SSI are satisfied, the biological data is stored on the blockchain-related medium and can be accessed by anyone,which means the user’s private information is easily leaked and lacks privacy. At the same time,the authentication can only be done by using a biometric extractor, which lacks flexibility. The literature [13] used DID and VC to design an eIDAS agent, implementing an SSI-based digital library identity management system, but it only applied to one system, lacking the flexibility of SSI. Reference [14] created a form of identity for WebID, which realized global identification and authentication in a distributed method by combining linked data and asymmetric encryption technology. Although the identity controllability of SSI is satisfied, both the public key and WebID are tightly bound to a configuration-oriented document describing authentication and stored in a third-party server like OpenID,which isn’t enough to decentralize SSI. Compared with the blockchain-based SSI system, Uport [15] has a similar function to Jolocom [16], which only allows to create one identity, so users cannot create an identity as needed, and does not support full autonomy of identity. Sovrin [17], whose main goal is to promote the concept of SSI certification, is still in the development cycle and lacks a method for fine-grained access control. HomeChain [19] upload its public key and its prepared group signature into the blockchain’s smart contract, implement ing anonymous security authentication, but it didn’t meet the fin-grained access control. It can be seen from the table that the proposed SDPP-SSI which supports privacy protection proposed in this scheme not only supports controllability, security and flexibility but also supports fine-grained access control and privacy protection.

**Table 7:** Comparison and analysis of the related work [12–19].

Literature	Controllability	Security	Flexibility	Privacy Protection	Authentication	Fine-grained Access Control
[12]	✓	✓	✕	✕	✓	✕
[13]	✓	✓	✕	✓	✓	✕
[14]	✕	✓	✕	✓	✓	✓
Uport [15]	✓	✓	✕	✕	✓	✕
Jolo [16]	✕	✓	✕	✕	✓	✕
Sovrin [17]	✓	✓	✓	✓	✓	✕
Blockcerts [18]	✓	✓	✕	✕	✓	✕
HomeChain [19]	✓	✓	✕	✓	✓	✕
The proposed SDPP-SSI	✓	✓	✓	✓	✓	✓

## Conclusion

First we propose a SDPP-SSI authentication framework, then we formally define the concept of SDPP-SSI identity through a mathematical model to classify identity management into traditional centralized and decentralized types. With the combination of federated chain and DID, DID is used as a global identifier to register, authenticate and revoke for user in decentralized domains. Finally, the VC is encapsulated using policy control signatures to implement the fine-grained access control, authentication, and revocation of user’s identity registration in the centralized domain. The proposed SDPP-SSI authentication protocol which not only meets controllability, authentication, and flexibility, but also supports privacy protection and fine-grained access control of identity information.

The proposed SDPP-SSI authentication protocol pays little attention to the change process for Identity information,it results in that the user needs to perform a cancellation process to change identity information. After that the user needs to complete the registration of a new identity through the registration process again, which increases the complexity of the system. Therefore, the next step is to study how to improve the identity modification process on an SSI authentication protocol that supports privacy protection.

## References

[1] Shuaib M, Alam S, Shahnawaz M, et al. Immunity credentials using self-sovereign identity for combating COVID-19 pandemic[J]. Materials Today: Proceedings, 2021. 10.1016/j.matpr.2021.03.096 PMC7983450 33777707

[2] Freytsis M, Barclay I, Radha S K, et al. Development of a Mobile, Self-Sovereign Identity Approach for Facility Birth Registration in Kenya[J]. Frontiers in Blockchain, 2021,4:2.

[3] Grech A, Sood I, Ariño L. Blockchain, Self-Sovereign Identity and Digital Credentials: Promise Versus Praxis in Education[J]. Frontiers in Blockchain, 2021,4:7.

[4] Wood G. Ethereum: A secure decentralised generalised transaction ledger[J]. Ethereum project yellow paper, 2014, 151(2014): 1–32.

[5] Androulaki E, Barger A, Bortnikov V, et al. Hyperledger fabric: a distributed operating system for permissioned blockchains[C]//Proceedings of the thirteenth EuroSys conference. 2018: 1-15.

[6] Gräther W, Kolvenbach S, Ruland R, et al. Blockchain for education: lifelong learning passport[C]//Proceedings of 1st ERCIM Blockchain workshop 2018. European Society for Socially Embedded Technologies (EUSSET), 2018.

[7] Soltani R, Nguyen U T, An A. A new approach to client onboarding using self-sovereign identity and distributed ledger[C]//2018 IEEE International Conference on Internet of Things (iThings) and IEEE Green Computing and Communications (GreenCom) and IEEE Cyber, Physical and Social Computing (CPSCom) and IEEE Smart Data (SmartData). IEEE, 2018: 1129-1136.

[8] Abraham A, Schinnerl C, More S. SSI Strong Authentication using a Mobile-Phone based Identity Wallet reaching a High Level of Assurance[C]//Proceedings-8th International Conference on Security and Cryptography (SECRYPT 2021). 2021.

[9] Aruna M G, Hasan M K, Islam S, et al. Cloud to cloud data migration using self sovereign identity for 5G and beyond[J]. Cluster Computing, 2021: 1–15. 10.1007/s10586-021-03461-7 PMC8591597 34803477

[10] Figueroa-Lorenzo S, Añorga Benito J, Arrizabalaga S. Modbus access control system based on SSI over hyperledger fabric blockchain[J]. Sensors, 2021, 21(16): 5438. doi: 10.3390/s21165438 34450880 PMC8398114

[11] Xurui Z, Fuyong Z, Xianming L, et al. A secure and policy-controlled signature scheme with strong expressiveness and privacy-preserving policy[J]. IEEE Access, 2021, 9:14945–14957. doi: 10.1109/ACCESS.2021.3052463

[12] Lin C., He D. B., Huang X. Y. and et al., “BSeIn: A blockchain-based secure mutual authentication with fine-grained access control system for industry 4.0,” Journal of Network and Computer Applications, 2018, pp. 42–52. doi: 10.1016/j.jnca.2018.05.005

[13] Zhu X. Y. and Youakim B., “A survey on blockchain-based identity management systems for the Internet of Things,” in 2018 IEEE International Conference on Internet of Things (iThings) and IEEE Green Computing and Communications (GreenCom) and IEEE Cyber, Physical and Social Computing (CPSCom) and IEEE Smart Data (SmartData), IEEE, 2018, pp. 1568-1573.

[14] Stokkink Q., Johan P., “Deployment of a blockchain-based SSI” in 2018 IEEE international conference on Internet of Things (iThings) and IEEE green computing and communications (GreenCom) and IEEE cyber, physical and social computing (CPSCom) and IEEE smart data (SmartData), IEEE, 2018, pp. 1336-1342.

[15] Hammudoglu J-S, Sparreboom J, Rauhamaa J-I and et al, “Portable trust: biometric-based authentication and blockchain storage for SSI systems,” arXiv preprint arXiv, 1706.03744, 2017.

[16] Othman A, Callahan J. The Horcrux protocol: a method for decentralized biometric-based self-sovereign identity[C]//2018 international joint conference on neural networks (IJCNN). IEEE, 2018: 1-7.

[17] Abraham A., Kevin T. and Emanuel K., “Qualified eID derivation into a distributed ledger based IdM system” in 2018 17th IEEE International conference on trust, security and privacy in computing and communications, IEEE, 2018, pp. 1406-1412.

[18] Guan Z, Garba A, Li A, et al. AuthLedger: A Novel Blockchain-based Domain Name Authentication Scheme[C]//ICISSP. 2019: 345-352.

[19] Lin C, He D, Kumar N, et al. HomeChain: A blockchain-based secure mutual authentication system for smart homes[J]. IEEE Internet of Things Journal, 2019, 7(2): 818–829. doi: 10.1109/JIOT.2019.2944400

[20] Cameron K., “The laws of identity,” Microsoft Corp, 2005, pp. 8–11.

[21] Isaak J. and Mina-J H., “User data privacy: facebook, cambridge analytica, and privacy protection,” Computer, vol. 51, no. 8, pp. 56–59, 2018. doi: 10.1109/MC.2018.3191268

[22] Allen C., “The Path to Self-Sovereign Identity” in Life with Alacrity, 2016,[Online]. Available: https://www.techscience.com/books/mlpg_atluri.html.

[23] Faísca J G, Rogado J Q. Decentralized semantic identity[C]//Proceedings of the 12th International Conference on Semantic Systems. 2016: 177-180

[24] Lundkvist C., Rouven H., T. Joel and et al, Uport: A platform for SSI. 2017 [Online]. Available: https://whitepaper.uport.me/uPort_whitepaper_DRAFT20170221.pdf.

[25] Fei C., Lohkamp J., Rusu E., Szawan K., K. Wagner and Wittenberg N., “Jolocom: Self-sovereign and decentralised identity by design”, 2018, [online] Available: https://tinyurl.com/v2ly5zx.

[26] Reed D., J. Law and Hardman D.,The Technical Foundations of Sovrin,2016, [online] Available: https://www.evernym.com/wp-content/uploads/2017/07/The-Technical-Foundations-of-Sovrin.pdf.

[27] Capece G., Ghiron Nathan L. and Francesco P., “Blockchain technology: redefining trust for digital certificates,” Sustainability, 2020. doi: 10.3390/su12218952

[28] Maria A, Pandi V, Lazarus J D, et al. BBAAS: Blockchain-based anonymous authentication scheme for providing secure communication in VANETs[J]. Security and Communication Networks, 2021, 2021. doi: 10.1155/2021/6679882

[29] Liu J, Li X, Jiang Q, et al. Bua: A blockchain-based unlinkable authentication in vanets[C]//ICC 2020-2020 IEEE International Conference on Communications (ICC). IEEE, 2020: 1-6.

[30] Gupta B B, Li K C, Leung V C M, et al. Blockchain-assisted secure fine-grained searchable encryption for a cloud-based healthcare cyber-physical system[J]. IEEE/CAA Journal of Automatica Sinica, 2021, 8(12): 1877–1890. doi: 10.1109/JAS.2021.1004003

[31] Nguyen G N, Le Viet N H, Elhoseny M, et al. Secure blockchain enabled Cyber–physical systems in healthcare using deep belief network with ResNet model[J]. Journal of parallel and distributed computing, 2021, 153: 150–160. doi: 10.1016/j.jpdc.2021.03.011

[32] Lu J, Shen J, Vijayakumar P, et al. Blockchain-based secure data storage protocol for sensors in the industrial Internet of Things[J]. IEEE Transactions on Industrial Informatics, 2021, 18(8): 5422–5431. doi: 10.1109/TII.2021.3112601

[33] Ferdous M., Farida C. and Madini-O A., “In search of SSI leveraging blockchain technology,” IEEE access, 2019, pp. 103059–103079. doi: 10.1109/ACCESS.2019.2931173

[34] Blanchet B., “An efficient cryptographic protocol verifier based on prolog rules,” in Citeseer, 2001, pp. 82–96.

